# Structural Landscape and Proton Conduction of Lanthanide 5-(Dihydroxyphosphoryl)isophthalates

**DOI:** 10.1021/acs.cgd.4c00786

**Published:** 2024-09-12

**Authors:** Inés R. Salcedo, Montse Bazaga-García, Rosario M. Pérez Colodrero, Álvaro Vílchez-Cózar, Fernando Cañamero-Cebrián, Pascual Olivera Pastor, Jan K. Zaręba, Aurelio Cabeza

**Affiliations:** † Departamento de Química Inorgánica, Universidad de Málaga, Campus Teatinos s/n, Málaga 29071, Spain; ‡ Servicios Centrales de Apoyo a la Investigación, Universidad de Málaga, Málaga 29071, Spain; § Institute of Advanced Materials, Faculty of Chemistry, Wrocław University of Science and Technology, Wrocław 50-370, Poland

## Abstract

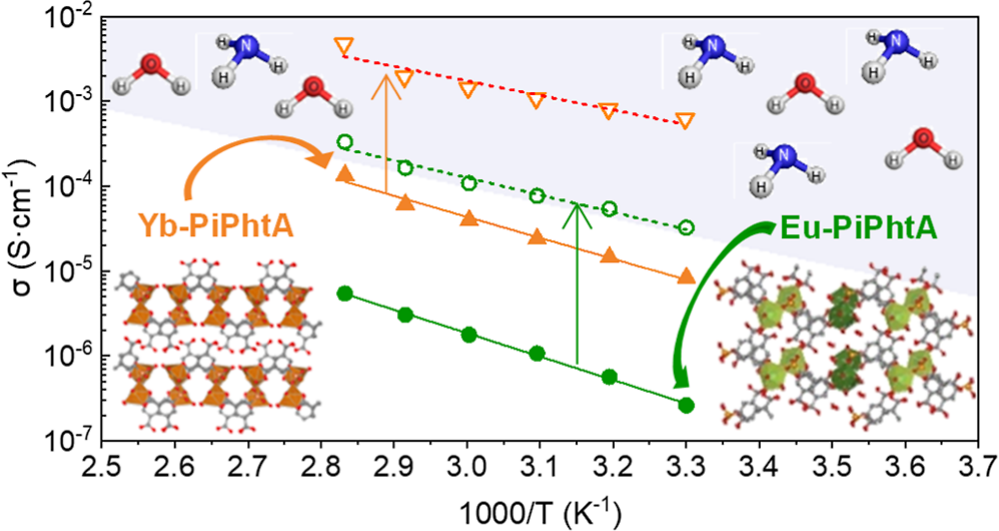

Metal phosphonate-carboxylate compounds represent a promising class of materials for proton conduction applications. This study investigates the structural, thermal, and proton conduction properties of three groups of lanthanide-based compounds derived from 5-(dihydroxyphosphoryl)isophthalic acid (PiPhtA). The crystal structures, solved *ab initio* from X-ray powder diffraction data, reveal that groups **Ln-I**, Ln[O_3_P–C_6_H_3_(COO)(COOH)(H_2_O)_2_] (Ln = La, Pr), and **Ln-II**, Ln_2_{[O_3_P–C_6_H_3_(COO)(COOH)]_2_(H_2_O)_4_}·2H_2_O (Ln = La, Pr, Eu), exhibit three-dimensional frameworks, while group **Ln-III**, Ln[O_3_P–C_6_H_3_(COO)(COOH)(H_2_O)] (Ln = Yb), adopts a layered structure with unbonded carboxylic groups oriented toward the interlayer region. All compounds feature carboxylic groups and coordinating water molecules. Impedance measurements demonstrate that these materials exhibit water-mediated proton conductivity, initially following a vehicle-type proton-transfer mechanism. Upon exposure to ammonia vapors from a 14 or 28% aqueous solution, compounds from groups **II** and **III** adsorb ammonia and water, leading to an enhancement in proton conductivity consistent with a Grotthuss-type proton-transfer mechanism. Notably, group **II** of the studied compounds undergoes the formation of a new expanded phase through the internal reaction of carboxylic groups with ammonia, coexisting with the as-synthesized phase. This postsynthetic modification results in a significant increase in proton conductivity, from approximately ∼5 × 10^–6^ to ∼10^–4^ S·cm^–1^ at 80 °C and 95% relative humidity (RH), attributed to a mixed intrinsic/extrinsic contribution. Remarkably, the NH_3_(28%)-exposed **Yb-III** compound achieves an enhancement in proton conductivity, reaching ∼ 5 × 10^–3^ S·cm^–1^ at 80 °C and 95% RH, primarily through an extrinsic contribution.

## Abstract 2

3D and 2D acidic lanthanide 5-(dihydroxyphosphoryl)isophthalates: proton conductivity and postsynthesis NH_3_/H_2_O adsorption.

## Introduction

Coordination polymers (CPs) are structurally versatile compounds with a great potential for a wide range of applications, among others, in catalysis, gas storage, ion exchange, medicine, and proton conductivity.^1−6^ Compared to metal–organic frameworks (MOFs), metal phosphonates represent a diverse subset of CPs exhibiting typically less porous structures. This difference arises from the nature of the phosphonate ligands, which tend to form stronger and more rigid bonds with metal ions.^7^ Attempts to generate metal-phosphonate-based open-framework structures, thus combining the characteristics of crystalline microporous materials with the specific features of metal phosphonates, mainly their robustness, have been carried out. Nevertheless, only the use of a few ligands, such as certain tritopic phosphonoaromatic ligands, has been proven successful for this purpose.^8^ Although the metal phosphonates frameworks are often more compact and less open than MOFs, their high stability coupled with the ability of the phosphonate group to coordinate also as protonated species, make them attractive as proton conducting materials.^9^

Lanthanide-based metal phosphonates have garnered significant attention due to the unique properties conferred by lanthanide elements. Recent comprehensive reviews^10,11^ have underscored the peculiar magnetic,^12−15^ luminescent,^16−19^ and catalytic properties^20−22^ of these materials, highlighting their potential in various applications.

The structural diversity of lanthanide phosphonates stems from the coordination flexibility of both lanthanide ions and phosphonate-based ligands.^8,23^ Lanthanide ions, characterized by their high coordination numbers, can form coordination complexes with diverse geometries, leading to the formation of structures with variable dimensionality, ranging from discrete clusters to 3D architectures.^12,24−29^ In addition, phosphonate-based ligands offer multiple coordination modes, facilitating the construction of intricate architectures through metal–ligand interactions.^30−32^ Moreover, by judiciously designing the structure of metal phosphonates, the luminescent and magnetic properties can be adequately modulated paving the way for applications in lighting, sensing, display technologies, or novel magnetic materials for data storage and spintronics.^33−35^

On the other hand, proton conduction in lanthanide metal phosphonates is primarily mediated by hydrogen-bond networks, in part owing to the ability of phosphonate groups to interact with lattice and coordinating water molecules. Given the structural versatility of lanthanide metal phosphonates the formation of extensive hydrogen-bonded networks is widespread, which in turn predisposes these materials for the efficient proton transport.^36−38^ The high coordination numbers and coordination flexibility of lanthanide ions further augment proton transport capabilities of these networks.^39,40^ Moreover, recent studies have emphasized that the incorporation of hydrogen-bond-forming guest molecules into the framework of lanthanide metal phosphonates significantly boosts their proton conductivity. This enhancement is attributed to the increased availability of proton carriers and the establishment of dynamic hydrogen-bond networks that facilitate proton hopping mechanisms.^41,42,45^ The thermal stability of lanthanide metal phosphonates is another vital aspect that influences their proton conductivity. These materials exhibit robust thermal stability, which is essential for their application in high-temperature proton exchange membrane fuel cells. The ability to maintain structural integrity and conductivity at elevated temperatures positions lanthanide metal phosphonates as promising candidates for such applications.^39,44^ The ligand of interest is a unique phosphonate/carboxylate hybrid, distinguishing it from purely tricarboxylic or triphosphonic acid derivatives. This type of dual-function ligand has been used to develop compounds with a wide structural diversity^45^ and proton conductivity (Table S1).^46−48^

In this study, we extend our investigation of the 5-(dihydroxyphosphoryl)isophthalic acid (PiPhtA)^49^ to the synthesis of lanthanide-based materials, with a particular focus on their proton conductivity properties. We explore postsynthetic modifications to enhance these properties, aiming to deepen our understanding of their conductive behavior through systematic analysis.

## Experimental Section

### Materials and Common Instrumentation

All chemical reagents were purchased from commercial sources and were used as received without further purification. The synthesis of 5-(dihydroxyphosphoryl)isophthalic acid (PiPhtA) has been previously described.^49^ Hydrated lanthanide nitrate or chloride reagents were purchased from Sigma-Aldrich. Stock solutions of NaOH 0.1 and 1 M were used for the pH adjustment. Ammonia (NH_3_, 28%) solution was purchased from VWR Chemicals. Deionized (DI) water was used for all of the syntheses. Elemental analyses were measured on a TruSpec Macro CHN/CHNS analyzer. Thermogravimetric analysis (TGA) data were recorded on an SDT-Q600 analyzer (TA Instruments) from RT to 900 °C at a heating rate of 10 °C/min. Measurements were carried out on samples in open platinum crucibles under an air flow.

### Synthesis

61 mg (0.25 mmol) of 5-(dihydroxyphosphoryl)isophthalic acid was dissolved in 10 mL of DI water. Separately, 0.25 mmol of the corresponding hydrated lanthanide nitrate or chloride was dissolved in 5 mL of DI water. The lanthanide solution was then added to the ligand solution, resulting in the formation of a precipitate. The final pH was adjusted to 3.0 using 0.1 and 1 M NaOH stock solution. The resulting mixture was transferred to a Teflon-lined autoclave and heated at 140 °C for 4 days, followed by cooling overnight. The precipitated solid was isolated by filtration, washed with DI, and dried at RT. Three groups of compounds **Ln-I** (La and Pr), **Ln-II** (La, Pr, and Eu), and **Ln-III** (Yb) were isolated. Elemental analysis (wt %) and representative formulas for the three sets of compounds are: La[O_3_P–C_6_H_3_(COO)(COOH)(H_2_O)_2_] **(Ln-I)** [Calc.: C 22.93, H 2.16; Found: C 22.11, H 1.7]; Eu_2_{[O_3_P–C_6_H_3_(COO)(COOH)]_2_(H_2_O)_4_}·2H_2_O **(Ln-II)** [Calc.: C 21.35, H 2.46; Found: C 20.96, H 2.26]; Yb[O_3_P–C_6_H_3_(COO)(COOH)(H_2_O)] (**Yb-III**) [Calc.: C 19.92, H 1.43; Found: C 20.20, H 1.52].

### Structural Characterization from Powder Diffraction Data

Synchrotron X-ray powder diffraction data for **La-I**, **Eu-II**, and **Yb-III** were collected at the BL04-MSPD beamline of ALBA (Barcelona, Spain). A wavelength of 0.6188 or 0.41318 Å was selected using a double-crystal Si(111) monochromator, determined from a Si640d NIST standard (*a* = 5.43123 Å) measurements, and data were collected using an MYTHEN detector. Partial structural models were obtained by the simulated annealing method using the program EXPO2014,^50^ and the positions of missing atoms corresponding to water molecules were localized by difference Fourier maps. All crystal structures were optimized by the Rietveld method^51^ using GSAS program^52^ and the graphic interface EXPGUI.^53^ For the remaining synthesized lanthanide 5-phosphonoisophthalates, laboratory X-ray powder diffraction data (LXRPD) were collected on a D8 ADVANCE (Bruker AXS) diffractometer using MoK_α1_ radiation (**Pr-I**), or an X’Pert MPD PRO diffractometer (PANalytical B.V.) and CuK_α1_ radiation (**La-II** and **Pr-II**), and their crystal structures were determined by Rietveld refinement from **La-I** or **Eu-II**, respectively. Soft constraints were used to maintain chemically reasonable geometries for the phosphonate, carboxylic/carboxylate, and aromatic ring groups. Hydrogen atoms were not located due to the limited quality of the XRPD data. Crystallographic data are presented in Table 1, and the final Rietveld plots are given in Figures S1–S6.

**Table 1:** Crystallographic Data for **Ln-I**, **Ln-II**, and **Yb-III** Series

	**La-I**	**Pr-I**^a^	**La-II**	**Pr-II**	**Eu-II**	**Yb-III**
empirical formula	LaC_8_O_9_PH_8_	PrC_8_O_9_PH_8_	La_2_C_16_O_20_P_2_H_20_	Pr_2_C_16_O_20_P_2_H_20_	Eu_2_C_16_O_20_P_2_H_20_	YbC_8_O_8_PH_6_
F.W. (g·mol^–1^)	418.02	420.02	872.07	876.08	898.19	434.14
space group	*Pbn*2_1_	*Pbn*2_1_	*P*1̅	*P*1̅	*P*1̅	*P*2_1_/*c*
λ (Å)	0.6188	0.7093	1.5406	1.5406	0.41318	0.41318
*a* (Å)	12.81071(8)	12.7611(5)	19.3334(11)	19.23273(16)	19.0566(4)	13.32953(17)
*b* (Å)	11.92582(7)	11.8466(4)	9.2969(6)	9.26913(10)	9.21439(18)	12.56039(16)
*c* (Å)	7.24072(4)	7.16845(22)	6.9856(7)	6.93650(25)	6.89011(12)	6.57557(10)
α (deg)	90.0	90.0	90.206(9)	90.1147(29)	89.5776(32)	90.0
β (deg)	90.0	90.0	93.066(9)	92.9539(27)	92.6910(23)	103.1900(14)
γ (deg)	90.0	90.0	94.148(4)	94.0787(8)	93.9222(16)	90.0
*V* (Å^3^)	1106.224(14)	1083.70(8)	1250.47(18)	1231.78(5)	1205.69(5)	1071.865(29)
*Z*	4	4	2	2	2	4
2θ range (deg)	0.37–43.2	3.88–50.0	4.0–69.9	7.0–119.98	0.37–42.5	0.37–42.5
data/restrains/parameters	7138/48/107	2307/47/105	2538/110/189	6647/110/191	7020/109/170	7020/57/113
no. reflections	1063	882	1077	3101	9868	6049
*R*_wp_	0.0800	0.0522	0.0916	0.0462	0.0796	0.0892
*R*_P_	0.0557	0.0372	0.0709	0.0338	0.0561	0.0614
*R*_F_	0.049	0.0957	0.0429	0.0423	0.0283	0.0229
CCDC number	2358821	2358822	2358818	2358819	2358820	2358817

a With minor impurities.

Thermodiffractometric studies were carried out using an Anton Paar TTK1200N camera under static air conditions. Samples were heated at selected temperatures from RT to 300 °C, depending on the compound, with a delay time of 15 min to ensure thermal equilibration.

### Gas Adsorption

N_2_ adsorption–desorption isotherms were obtained using the Micromeritics ASAP 2020 (USA) surface area analyzer at 77 K at high vacuum. The samples were degassed at 150 °C for at least 12 h prior to the measurement. Water vapor sorption isotherms were measured at 25 °C. Before each sorption measurement, the samples were activated under vacuum at 150 °C for 2 h.

NH_3_-exposed derivatives were prepared as elsewhere reported.^49,54,55^**Eu-II** and **Yb-III** were exposed to ammonia vapors from 14% NH_3_ aqueous solution in a closed container for a maximum of 12 days and without any previous treatment. **Yb-III** was also exposed to ammonia vapors from a 28% NH_3_ aqueous solution for 4 days. Elemental analyses (wt %) for ammonia derivatives: Eu_2_(C_8_H_4_PO_7_)_2_(H_2_O)_8.5_(NH_3_)_1.15_ (**Eu-II-NH**_**3**_**-14%**), Calc.: C 19.84, H 3.07, N 2.17; Found: C 19.81, H 2.55, N 2.15. For Yb(C_8_H_4_PO_7_)(H_2_O)_3.5_(NH_3_)_0.45_ (**Yb-III-NH**_**3**_**-14%**), Calc.: C 19.73, H 2.55, N 1.29; Found: C 19.84, H 2.15, N 1.27. For Yb(C_8_H_4_PO_7_)(H_2_O)_3.5_(NH_3_)_0.8_ (**Yb-III-NH**_**3**_**-28%**), Calc.: C 19.45, H 2.74, N 2.27; Found: C 19.68, H 2.73, N 2.55. **Eu-II-NH**_**3**_**-14%**, **Yb-III-NH**_**3**_**-14%**, and **Yb-III-NH**_**3**_**-28%** were re-exposed to 28% NH_3_ aqueous solution for 12 h. Elemental analyses (wt %) for NH_3_ (28%) reloaded samples: Eu_2_(C_8_H_4_PO_7_)_2_(H_2_O)_7_(NH_3_) (**Eu-II-NH**_**3**_**-28%_R**), Calc.: C 20.54, H 2.69, N 1.50; Found: C 20.32, H 2.81, N 1.43; Yb(C_8_H_4_PO_7_)(H_2_O)_2_(NH_3_)_0.3_ (**Yb-III-NH**_**3**_**-28%_R**), Calc.: C 20.56, H 2.14, N 0.90 Found: C 20.15, H 2.12, N 0.94.

### Proton Conductivity

Impedance measurements were carried out on cylindrical pellets (diameter ∼5 mm and thickness ∼1 mm) obtained by pressing ∼40 mg of sample at 250 MPa for 1 min. Pellets were painted with silver ink and placed between porous carbon electrodes (Sigracet, GDL 10 BB, no Pt) inside a temperature- and humidity-controlled chamber (Espec SH-222). EIS data were acquired with an AUTOLAB PGSTAT302N impedance analyzer over the frequency range from 20 Hz to 1 MHz with an applied voltage of 0.35 V. The pellets were first preheated (0.2 °C·min^–1^) from 25 to 80 °C and 95% relative humidity (RH) to ensure equilibrium with the atmosphere, and EIS data were recorded on cooling using a stabilization time of 5 h at each measured temperature. To prevent water condensation on the samples, the RH was reduced before decreasing the temperature. The measurements were automatically controlled with NOVA 2.1.7 software.^56^ For all compounds, the total pellet resistance (R_T_) was obtained from the analysis of the spectra using the ZView program.^57^

## Results and Discussion

Under essentially similar experimental conditions, three types of structures have been obtained. As below discussed, the formation of the frameworks strongly depends on the size of lanthanide ions: 3D structures is favored for highly coordinated large ions (La^3+^ – Eu^3+^), while a layered structure is favored for the heavier seven-coordinated Yb^3+^ ion.

### Crystal Structure of Ln[O_3_P–C_6_H_3_(COO)(COOH)(H_2_O)_2_] (Ln-I, Ln = La and Pr)

**Figure 1:**
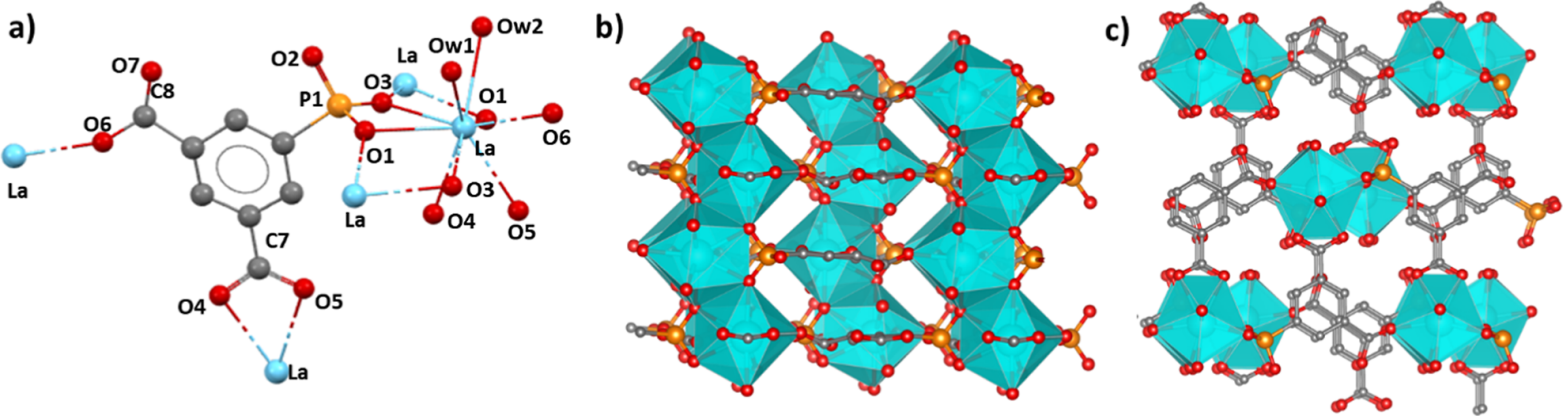
Structural details for La[O_3_P–C_6_H_3_(COO)(COOH)(H_2_O)_2_] (**La-I)**: (a) extended asymmetric unit, (b) chains of edge-sharing LaO_9_ running along the *c*-axis (viewed along the *b*-axis), and (c) connection of the LaO_9_ chains viewed from the *c*-axis (*b*-axis in horizontal).

The compounds of the isostructural subgroup **Ln-I** crystallize in the orthorhombic system with space group *Pbn*2_1_. Structural details are discussed for the La^3+^ derivative as a representative example of group **I**. The asymmetric unit consists of one La^3+^ ion, one PiPhtA^3–^ ion, and two coordinating water molecules. The nine-coordinated environment of La^3+^ ions is composed of seven oxygen atoms, from phosphonate and the carboxylic and carboxylate groups of four distinct ligands, and two water molecules (Figure 1a). Notably, the phosphonate O2 and carboxylic O7 remain unbonded; thus, one carboxylic group, involved in the formation of H-bonds, and one carboxylate group, acting as chelate group, are present in this framework. The structure is built up of chains of edge-sharing LaO_9_ polyhedra, running along the *c*-axis, interconnected through the phosphonate and carboxylate groups (Figure 1b,c), with each ligand linking three chains. According with donor–acceptor distances (Table S2), the unbonded O2 is interacting by a H-bond with Ow1 [2.97(4) Å] and Ow2 [2.88(4) Å] and O7 [2.46(2) Å] from the monodentate carboxylic group.

### Crystal Structure of Ln_2_{[O_3_P–C_6_H_3_(COO)(COOH)]_2_(H_2_O)_4_}·2H_2_O (Ln-II, Ln = La, Pr, and Eu)

**Figure 2:**
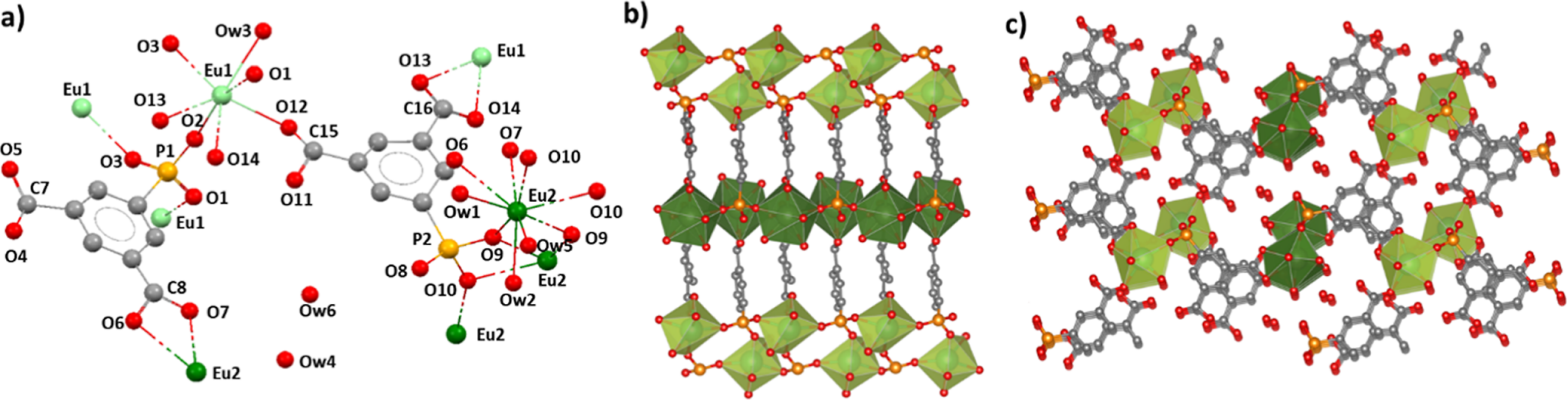
Structural details for Eu_2_{[O_3_P–C_6_H_3_(COO)(COOH)]_2_(H_2_O)_4_}·2H_2_O **(Eu-II)**: (a) extended asymmetric unit, (b) packing of alternating edge-sharing Eu2O_9_ (dark green) and isolated Eu1O_7_ (light green) chains, running along the *c*-axis (viewed along the *b*-axis), and (c) connectivity between Eu2O_9_ and Eu1O_7_ chains (viewed along the *c*-axis and *a*-axis in horizontal).

Compounds of the isostructural set **Ln-II** crystallize in the triclinic system, space group *P*1̅. The Eu^3+^ derivative, discussed as a representative example, contains in the asymmetric unit 40 non-H atoms with two crystallographically independent Eu^3+^ ions (Eu1 and Eu2), two PiPhtA^3–^ ions (L1 and L2), and six water molecules. Eu1 is hepta-coordinated, involving three oxygen atoms (O1, O2, and O3) from three different phosphonate groups, three oxygen atoms from one carboxylic (O12), a second carboxylate (O13 and O14) group, and one water molecule (Ow3) (Figure 2a). Eu2, on the other hand, is nine-coordinated by four oxygen atoms (O9, O9, O10, and O10), from three different phosphonate groups, the oxygen atoms (O6 and O7) of a carboxylate group, and three water molecules (Ow1, Ow2, and Ow5). The 3D crystal structure can be described as being composed of alternating chains of edge-sharing Eu2O_9_ polyhedra and isolated Eu1O_7_ polyhedra (Figure 2b). Both types of chains are distinctively interconnected by ligands L1 and L2. Accordingly, ligand L1 bridges monodentately three Eu1O_7_ polyhedra through the phosphonate (P1) oxygens while chelating Eu2 through one of the carboxylate groups (C8). This leaves a free carboxylic group (C7). L2 ligand bridges and chelates Eu2, through O9 and O10 of P2, leaving O8 unbonded, while chelating one Eu1 by one carboxylate group (C16) and coordinating another Eu1 in monodentate fashion, through O12 from C15. As shown in Table S3, the unbonded O5 and O11 interact by a H-bond with Ow3 [2.67(4) Å] and Ow4 [2.79(3) Å]; the latter also interacts by a H-bond with Ow1 [2.55(3)] Å, Ow2 [2.78(3) Å], Ow5 [2.42(4) Å], and Ow6 [2.93(4) Å], although no extended H-bond networks are formed.

**Figure 3:**
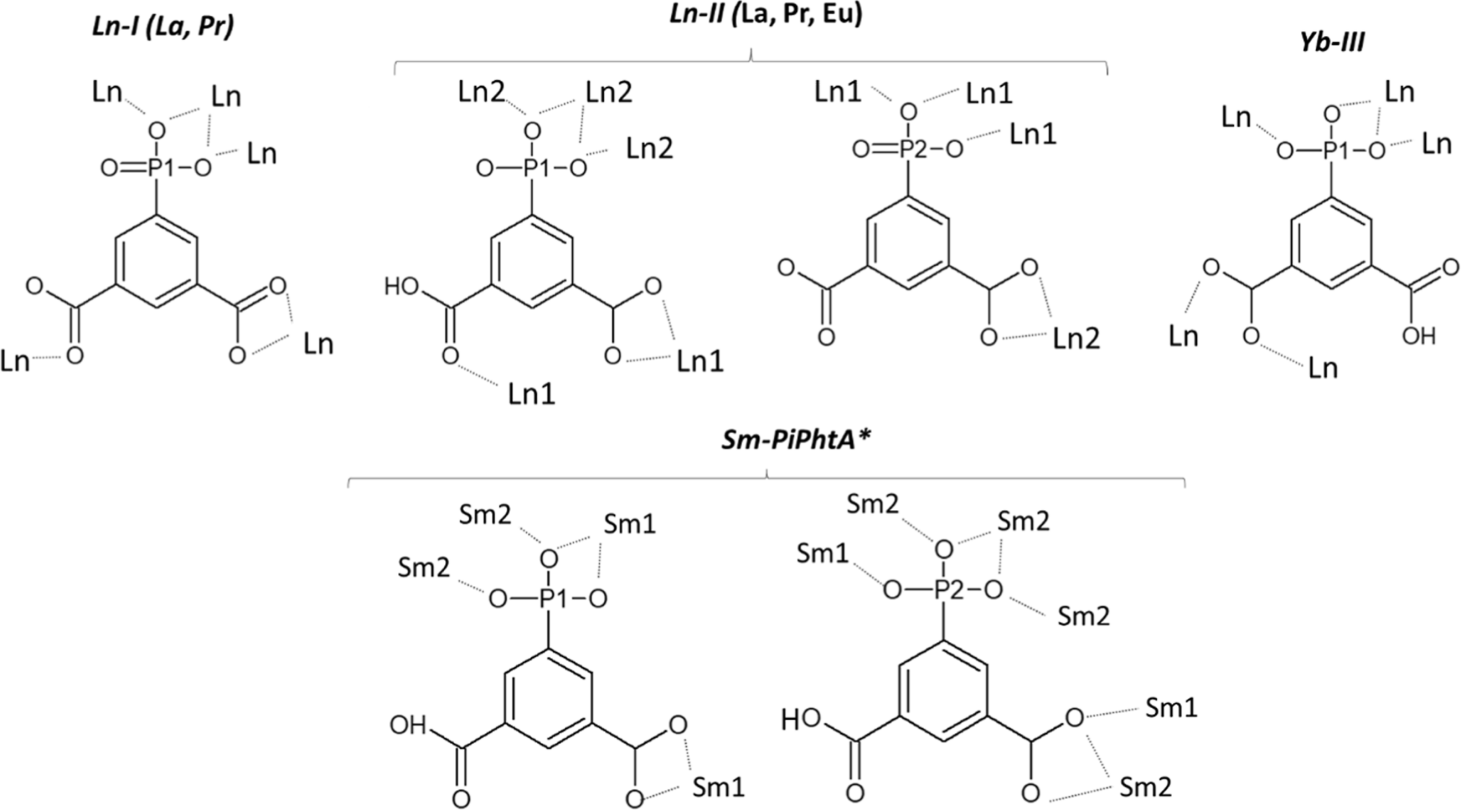
Coordination modes of the ligand PiPhtA in groups **Ln-I**, **Ln-II**, and **Ln-III**, compared with those in Sm-PiPhtA (adapted from ref (45)).

Compounds of group **II** represent polymorphic forms of the previously reported samarium derivative^45^ obtained under different synthesis conditions, which resulted in distinct metal–ligand connectivity motifs (Figure 3). The Sm^3+^ compound, Sm_2_{[O_3_P–C_6_H_3_(COO)(COOH)]_2_(H_2_O)_4_}·2H_2_O, exhibits a 2D structure featuring two types of nine-coordinated Sm^3+^ ions, one of which (Sm2) forms edge-sharing chains of SmO_9_ polyhedra, further connected to edge-sharing Sm1O_9_ side arms. In contrast, a different metal–ligand connectivity was attained from the reaction of Eu^3+^ with the more flexible ligand phosphonomethyl isophthalic acid,^43^ which gave rise to a layered anionic framework counterbalanced by (Me_2_NH_2_)^+^ cations intercalated in-between the layers. Remarkably, this structure facilitates the formation of strong hydrogen bond chains, assembled by the organic cations and the unbonded oxygen atoms from the phosphonate groups.

### Crystal Structure of Yb[O_3_P–C_6_H_3_(COO)(COOH)(H_2_O)] (Yb-III)

**Figure 4:**
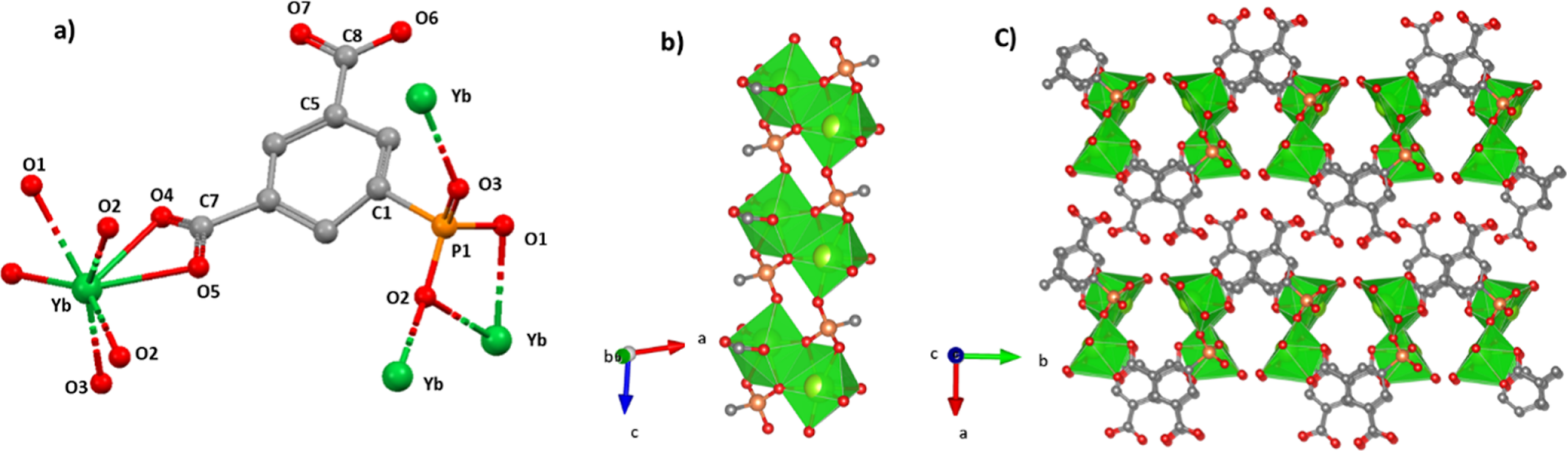
Structural details for Yb[O_3_P–C_6_H_3_(COO)(COOH)(H_2_O)] (**Yb-III**): (a) extended asymmetric unit, (b) view of the Yb_2_O_12_ chains running along the *c*-axis, and (c) layer packing along the *a*-axis.

This compound crystallizes in the monoclinic system space group *P*2_1_/*c*. The asymmetric unit (Figure 4a) consists of one hepta-coordinated Yb^3+^ ion, one PiPhtA^3–^ ion, and one coordination water molecule. The phosphonate group coordinates to three Yb^3+^ ions, chelating one Yb^3+^ ion via O1 and O2, the latter one also bridging another Yb^3+^ ion, while O3 binds to a third Yb^3+^ ion. This arrangement forms Yb_2_O_12_ dimers along the *c*-axis, where pairs of adjacent Yb^3+^ ions share two O2 atoms from two ligands and through two bridging carboxylate groups from another two ligands, resulting in each dimer being bonded to six ligands. The second carboxylate group is unbonded and protonated, pointing toward the interlayer region in an interdigitated manner, which creates H-bond interactions (Table S4) between the carboxylic O7 and the coordinating water molecule Ow1 of an adjacent layer [2.82(2) Å] and the carboxylic O6 and the carboxylate O4 [2.91(2) Å] (Figure 4b,c).

### FT-IR Spectroscopy

FT-IR spectra for selected compounds from each Ln-PiPhtA subgroup are depicted in Figures S7–S9. In addition to the characteristic P–O and P–C stretching vibration bands (900 and 1250 cm^–1^), signals corresponding to both carboxylic and carboxylate groups appear around 1700, 1600, and 1540 cm^–1^ (antisymmetric ν_C=O_) and around 1420 and 1360 cm^–1^ (symmetric ν_C–O_).^49^ The O–H stretching vibrations are identified between 3615 and 3274 cm^–1^. Specifically, the band at 3615 cm^–1^, which is present in the spectra of the compound of group **II**, can be attributed to bound water, while those in the range 3500–3274 cm^–1^, present in the spectra of the three groups of compounds, can be assigned to hydrogen bond-interacting water. No significant changes were detected in the FT-IR spectra of groups **I** and **III** were observed upon ammonia exposure. However, for **Yb-III**, an increased water content and the presence of a residual amount of ammonia, as determined by elemental analysis, indicate that the adsorption of NH_3_/H_2_O occurred (Figure S9). Conversely, for compounds of group **II,** broadening of the bands in the O–H stretching region, together with a remarkable decrease in intensity of the antisymmetric ν_C=O_ (1700 cm^–1^), suggest an extensive reaction between ammonia and the carboxylic groups (Figure S8).

### Proton Conductivity

**Figure 5:**
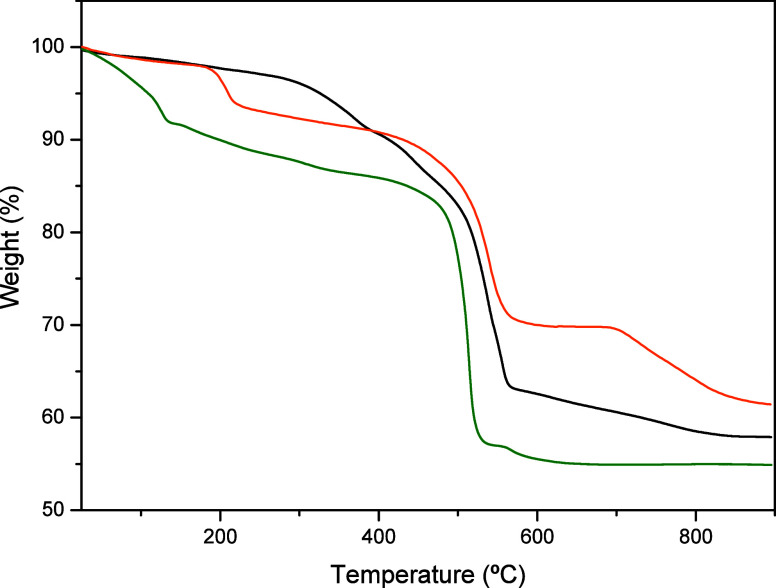
TGA curves for **La-I** (black), **Eu-II** (green), and **Yb-III** (orange) as representative derivatives of each group of compounds.

A preliminary survey by thermal analysis reveals appreciable differences relative to how strongly water is bound into the networks of the synthesized compounds (Figure 5). For group **I** compounds, Ln[O_3_P–C_6_H_3_(COO)(COOH)(H_2_O)_2_] (Ln = La and Pr), the TG curve shows a gradual weight loss between ∼150 and 400 °C consistent with the presence of only a single type of bound water to the lanthanide ions (observed: 8.47%; calculated: 8.60%). In contrast, group **II** compounds, Ln_2_{[(O_3_P–C_6_H_3_(COO)(COOH)]_2_(H_2_O)_4_]}·2H_2_O (Ln = La, Pr, and Eu), contain both lattice and coordinating water molecules. The former type is lost between 50 and 180 °C (observed: 8.59%; calculated: 8.0%), while the latter is smoothly lost from 180 up to ∼450 °C, contributing to the total weight loss of 11.96% (calculated: 12.01%). This solid can be partially dehydrated up to 180 °C and then reversibly rehydrated by exposure of the sample to high RH (98% in the presence of a saturated solution of K_2_SO_4_) (Figure S10). Yb[O_3_P–C_6_H_3_(COO)(COOH)(H_2_O)] (**Yb-III**) losses the bound water molecule between 150 and 270 °C (observed: 5.20%; calculated: 4.14%), and its rehydration is not reversible (Figure S11). Both groups of compounds, **Ln-II** and **Ln-III**, decompose at temperatures higher than 500 °C.

**Figure 6:**
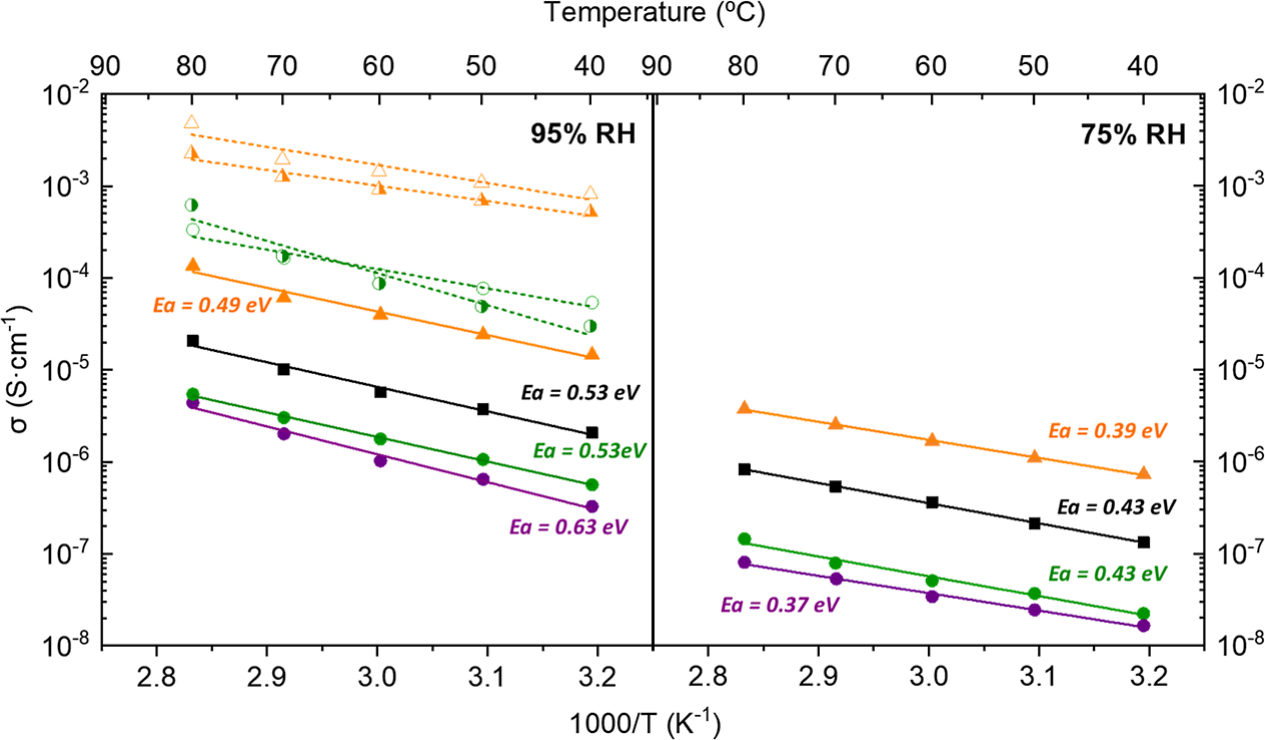
Arrhenius plots at 95% (left) and 75% RH (right) for **La-I** (square), **Ln-II** [circle; **La-II** (purple), and **Eu-II** (green)] and **Yb-III** (triangle) (closed symbol: as-synthesized, open symbol: **Eu-II-NH**_**3**_-**14%** and **Yb-III-NH**_**3**_**-28%**, half right symbol: **Eu-II-NH**_**3**_**-28%_R** and **Yb-III-NH**_**3**_**-28%_R**).

Given the presence of acidic groups, the proton conductivity of these solids was investigated. Nyquist and Arrhenius plots for representative derivatives are shown in Figures S12–S14 and 6, respectively. In all cases, proton conductivity increases with RH, indicating a water-mediated mechanism (Figure 6). The activation energies for the three groups of compounds (*E*_a_ = 0.48–0.63 eV) are consistent with a vehicle-type proton transport mechanism for most of cases,^58^ in accordance with the absence of extended H-bond networks as revealed by crystallographic data. The proton conductivity values at 80 °C and 95% RH, were 5.4 × 10^–6^, 2.1 × 10^–5^, and 1.3 × 10^–4^ S·cm^–1^, for **Eu-II**, **La-I**, and **Yb-III**, respectively. The postimpedance XRPD patterns (Figures S15 and S16) suggest no appreciable structural changes for Ln-PiPhtA compounds with respect to the as-synthesized solids.

Attempts to enhance proton conduction were made by exposing the samples to ammonia vapors (Table 2). As determined by elemental and thermal analyses, compounds of groups **II** and **III** adsorbed ammonia plus water (Figure S17). The XRPD patterns of both **Eu-II-NH**_**3**_**-14%** and **Yb-III-NH**_**3**_**-28%** (Figure S15) indicate a differential behavior on ammonia adsorption. For the europium derivative, a new poorly crystalline, expanded phase coexisted together with the as-synthesized phase, while the ytterbium derivative remained unchanged. In the case of the europium derivative, the indexing of the expanded phase was not possible. The peaks attributed to this phase appear at 10.97, 9.11, and 7.05 Å, which can be compared with the reflections (200), (010), and (001) at 9.393, 9.21, and 6.78 Å of the as-synthesis **Eu-II**, respectively. This suggests an expansion of the unit cell upon NH_3_/H_2_O adsorption, primarily along the *a*-axis and *c*-axis (Figure S15a). This behavior contrasts with that observed for other NH_3_-loaded metal phosphonates, where partial size particle-related amorphization^54^ or an evolution from the linker-pillared structure to a NH_3_/H_2_O-intercalated 2D framework were observed.^49^ These samples exhibit an enhancement of the proton conductivity values rising to 3.3 × 10^–4^ S·cm^–1^ (**Eu-II-NH**_**3**_**-14%**), 8.6 × 10^–4^ S·cm^–1^**(Yb-III-NH**_**3**_**-14%**), and 4.9 × 10^–3^ S·cm^–1^, and **(Yb-III-NH**_**3**_**-28%**), when measured at 80 °C and 95% RH (Figures S18 and S19), which are comparable with those reported for other metal phosphonate^49,54,55^ or isophthalates.^43^ Overall, these data suggest that the enhanced proton conduction of the NH_3_-exposed derivatives results from a mostly extrinsic contribution, i.e., that arising from interparticle transport, for the Yb^3+^ derivative, or from a mixed intrinsic/extrinsic contribution,^59^ for the Eu^3+^ derivative, with activation energy values being characteristics, in both cases, of a Grothuss-type proton-transfer mechanism (*E*_a_ ∼ 0.3–0.4 eV). The postimpedance XRPD patterns (Figures S15, S18 and S19) suggest no appreciable structural changes for Ln-PiPhtA compounds with respect to the as-synthesized solids. However, for **Eu-II**, desorption of adsorbed NH_3_ occurred under the EIS measurement conditions (Figure S19a). In our case, the internal inaccessibility to NH_3_ in 3D **Ln-I** and 2D **Ln-III** can be attributed to strong bonds impeding the access of guest molecules as basic as ammonia to the internal spaces of these structures. In fact, as determined by N_2_ adsorption, all the studied materials, including the NH_3_-loaded compounds, were essentially nonporous solids, with BET surface areas between 5.3 and 15.5 m^2^/g (Figures S20 and S21). In addition, water vapor isotherms indicate low water adsorption (Figures S22 and S23), although the adsorbed water tends to be strongly retained by the solids, except for **La-I**.

**Table 2:** Summary of Proton Conductivity (S·cm^–1^) and *E*_a_ (eV) Values for As-synthesized and NH_3_-Containing Compounds Measured at 80 °C and 95% RH

	as-synthesized compounds					
	**La-I**		**Eu-II**		**Yb-III**	
cycle of EIS measurements	σ	*E*_a_	σ	*E*_a_	σ	*E*_a_
1st	2.1 × 10^–5^	0.53	5.4 × 10^–6^	0.53	1.3 × 10^–4^	0.49

	NH_3_-loaded compounds					
	**Eu-II-NH**_**3**_**-14%**		**Yb-III-NH**_**3**_**-14%**		**Yb-III-NH**_**3**_**-28%**	
cycle of EIS measurements	σ	*E*_a_	σ	*E*_a_	σ	*E*_a_
1st	3.3 × 10^–4^	0.39	8.6 × 10^–4^	0.28	4.9 × 10^–3^	0.34
2nd	1.0 × 10^–4^	0.36	3.1 × 10^–4^	0.28	3.9 × 10^–4^	0.28
3rd	5.9 × 10^–5^	0.27	1.3 × 10^–4^	0.29	3.5 × 10^–4^	0.26
4th	4.7 × 10^–5^	0.27	1.1 × 10^–4^	0.28		
5th (NH_3_ (28%)-reloaded)^a^	6.2 × 10^–4^	0.69	1.3 × 10^–3^	0.41	2.3 × 10^–3^	0.33

a Samples from 4th circle exposed to NH_3_ (28%) vapors.

An interesting feature found in these materials is that a reversibly adsorption/desorption of NH_3_/H_2_O occurs, and thus, the proton conductivity is almost completely recovered upon readsorption of NH_3_/H_2_O after conducting various cycles of EIS measurements (Figures 6, S19 and Table 2), as determined by elemental and thermal analyses. Furthermore, it must be noted that for **Eu-II-NH**_**3**_**-14%**, its proton conductivity, after four cycles of EIS measurements, is still 1 order of magnitude higher than that for the as-synthesized **Eu-II**. However, although **Yb-III-NH_3_–28%_R** maintains a low activation energy (Table 2), typical of Grotthuss-type mechanism, the activation energy value for **Eu-II-NH_3_–28%_R** (*E*_a_ = 0.69 eV) increases, indicating a vehicle-type proton-transfer mechanism. The XRPD patterns for the NH_3_ (28%) reloaded samples after EIS measurements, Figure S15, display that the frameworks of both solids are maintained, which confirms the robustness of these frameworks as well as the important role of the extrinsic contribution to the proton conductivity. It is noteworthy that a gradual amorphization occurs in the europium derivative, which can be attributed to progressive particle size reduction.^54^ This is confirmed by SEM (Figure S24), which shows that the initial micrometer plate/blade particles of the as-synthesized compounds are transformed into aggregates of submicrometric particles.

## Conclusions

Three groups of lanthanide 5-(dihydroxyphosphoryl)isophthalates, with the general empirical formula Ln[O_3_P–C_6_H_3_(COO)(COOH)(H_2_O)_*x*_]·*n*H_2_O, namely, **Ln-I** (Ln^3+^ = La^3+^, Pr^3+^; *x* = 2), **Ln-II** (Ln^3+^ = La^3+^, Pr^3+^, Eu^3+^; *x* = 2, *n* = 1), and **Ln-III** (Ln^3+^ = Yb^3+^; *x* = 1) have been synthesized. They display distinct structural motifs based on the metal–ligand connectivity in such a way that 3D frameworks or a 2D network result. Specifically, **Ln-I** features ligand-interconnected edge-sharing LnO_9_ chains, while in **Ln-II** these edge-sharing LnO_9_ chains are sandwiched by isolated LnO_7_ chains. On the other hand, in **Yb-III**, the layers are built up by chains of isolated Yb_2_O_12_ dimers. The inherent acidity of these compounds, derived from carboxylic groups, prompted an investigation into the proton conductivity of the as-synthesized forms and those derivatized by exposure to ammonia vapors. The as-synthesized compounds exhibit moderate proton conductivities (5.4 × 10^–6^ < σ < 1.3 × 10^–4^ cm^–1^ for **Eu-II** and **Yb-III**, respectively, at 80 °C and 95% RH). While no ammonia adsorption occurred for **Ln-I**, only the outer acidic surface groups of **Yb-III** were accessible to the interaction with NH_3_. Conversely, **Ln-II** compounds exhibited a partial intercalation of NH_3_/H_2_O. In contrast to the as-synthesized compounds, the NH_3_-containing materials exhibited a Grothuss-type proton-transfer mechanism, which is attributed to the formation of extended H-bond networks. Thus, the best-performing proton conductor was **Yb-III-NH**_**3**_-**28%** with a value of ∼5 × 10^–3^ S·cm^–1^ at 80 °C and 95% RH. Interestingly, these materials undergo a reversible NH_3_/H_2_O adsorption/desorption process, achieving proton conductivity values similar to the initial ones.

## Supporting Information Available

All new structures and Rietveld plots for **Ln-I**, **Ln-II**, and **Ln-III** compounds; FT-IR spectrum for the as-synthesized and upon NH_3_-exposed samples; XRPD patterns after rehydration for selected samples; complex impedance plane plots for representative compounds of each series at 95% RH at different temperatures; XRPD patterns after impedance measurements; TG curves for NH_3_-containing samples; complex impedance plane plots for NH_3_-containing **Eu-II** and **Yb-III** at 95% RH and different temperatures; N_2_ and H_2_O vapor adsorption isotherms and SEM images for some representative as-synthesized and NH_3_-containing samples; structural features of metal di-, tricarboxylates, and triphosphonates; and H-bond interactions for representative compounds of each group (PDF)

## Abbreviations

PiPthA: 5-(dihydroxyphosphoryl)isophthalic acid
PEMFCs: proton exchange membrane fuel cells
LXRPD: laboratory X-ray powder diffraction
SXRPD: synchrotron X-ray powder diffraction
DI: deionized water
EIS: electrochemical impedance spectroscopy
ICP: inductively coupled plasma optical emission spectrometry
RT: room temperature
TGA: thermogravimetric analysis
