# Supplementary material for: Structural Landscape and Proton Conduction of Lanthanide 5-(Dihydroxyphosphoryl)isophthalates
Source: Cryst Growth Des. 2024 Sep 12;24(19):7910–8. doi: 10.1021/acs.cgd.4c00786 (PMC11450753; doi:10.1021/acs.cgd.4c00786)
Supplement: Supplementary file 1 — cg4c00786_si_001.pdf [file cg4c00786_si_001.pdf]

SUPPORTING INFORMATION

Structural landscape and proton conduction of lanthanide 5-(dihydroxyphosphoryl)isophtalates

Inés R. Salcedo[‡], Montse Bazaga-García[†], Rosario M. Pérez Colodrero[†], Álvaro Vílchez-Cózar[†], Fernando Cañamero-Cebrián[†], Pascual Olivera Pastor[†], Jan K. Zaręba[§,*] *and Aurelio Cabeza[†,*]*

[†] Departamento de Química Inorgánica, Universidad de Málaga, Campus Teatinos s/n, Málaga-29071, Spain.

[‡] Servicios Centrales de Apoyo a la Investigación, Universidad de Málaga, Málaga-29071, Spain.

[§] Institute of Advanced Materials, Faculty of Chemistry, Wrocław University of Science and Technology, 50-370 Wrocław, Poland.

Contents

Figure S1. Rietveld plot for $La[O_3P\text{-}C_6H_3(COO)(COOH)(H_2O)_2]$, **La-I**.

Figure S2. Rietveld plot for $Pr[O_3P\text{-}C_6H_3(COO)(COOH)(H_2O)_2]$, **Pr-I**.

Figure S3. Rietveld plot for $La_2\{[O_3P\text{-}C_6H_3(COO)(COOH)]_2(H_2O)_4\}\cdot 2H_2O$, **La-II**.

Figure S4. Rietveld plot for $Pr_2\{[O_3P\text{-}C_6H_3(COO)(COOH)]_2(H_2O)_4\}\cdot 2H_2O$, **Pr-II**.

Figure S5. Rietveld plot for $Eu_2\{[O_3P\text{-}C_6H_3(COO)(COOH)]_2(H_2O)_4\}\cdot 2H_2O$, **Eu-II**.

Figure S6. Rietveld plot for $Yb[O_3P\text{-}C_6H_3(COO)(COOH)(H_2O)]$, **Yb-III**.

Figure S7. FT-IR spectrum for as-synthesized **La-I**.

Figure S8. FT-IR spectra for as-synthesized **Eu-II** (black) and upon exposure to ammonia vapors, **Eu-II-NH$_3$-14%** (blue).

Figure S9. FT-IR spectra for as-synthesized **Yb-III** (black) and **Yb-III-NH$_3$-14%** (blue).

Figure S10. X-ray powder diffraction patterns for **Eu-II** compound as-synthesized (black), heated at 200 ºC (red) and rehydrated (blue) at RT.

Figure S11. X-ray powder diffraction patterns for **Yb-III** as-synthesized (black) and after heated at 300 ºC and rehydrated in K_2SO_4 saturated solution atmosphere at RT (blue).

Figure S12. Complex impedance plane plots for **La-I** at 95% RH and different temperatures: 80 (black), 70 (red), 60 (green), 50 (blue), 40 (cyan) and 30 ºC (magenta).

Figure S13. Complex impedance plane plots for (a) **La-II** and (b) **Eu-II** at 95% RH and different temperatures: 80 (black), 70 (red), 60 (green), 50 (blue), 40 (cyan) and 30 ºC (magenta).

Figure S14. Complex impedance plane plots for **Yb-III** at 95% RH and different temperatures: 80 (black), 70 (red), 60 (green), 50 (blue), 40 (cyan) and 30 ºC (magenta).

Figure S15. X-ray powder diffraction patterns for (a) **Eu-II** and (b) **Yb-III** compounds. * Corresponds to a new NH_3-containing **Eu-II** phase.

Figure S16. X-ray powder diffraction patterns for (a) **Eu-II** and (b) **Yb-III** compounds.

Figure S17. TG curves for **Eu-II-NH$_3$-14%** (solid green), **Eu-II-NH$_3$-28%_R** (dash dot green), **Yb-III-NH$_3$-14%** (solid orange), **Yb-III-NH$_3$-28%** (solid purple) and **Yb-III-NH$_3$-28%_R** (dash dot purple) derivatives.

Figure S18. Complex impedance plane plots at 95% RH for (a) **Eu-II-NH$_3$-14%**, (b) **Yb-III-NH$_3$-14%** and (c) **Yb-III-NH$_3$-28%** at different temperatures: 80 (black), 70 (red), 60 (green), 50 (blue), 40 (cyan) and 30 ºC (magenta).

Figure S19. Arrhenius plots for NH_3-containing derivatives at 95% RH and different EIS measurements: (a) **Eu-II** as-synthetized (●), **Eu-II-NH$_3$-14%** 1st (○), 2nd (⦶), 3rd (⊕) and 4rd cycles (◒); (b) **Yb-III** as-synthetized (▲), **Yb-III-NH$_3$-14%** 1st (△), 2nd (△), 3rd (△) and 4rd cycles (▲); (c) **Yb-III** as-synthetized (▲), **Yb-III-NH$_3$-28%** 1st (△), 2nd (△) and 3rd cycles (▲).

Figure S20. N_2 adsorption isotherms for: (a) La[O_3P-C_6H_3(COO)(COOH)(H_2O)$_2$] (**La-I**), (b) Eu_2{[O_3P-C_6H_3(COO)(COOH)]$_2$(H_2O)$_4$}·2H_2O **(Eu-II)** and (c) Yb[O_3P-C_6H_3(COO)(COOH)(H_2O)] **(Yb-III)**.

Figure S21. N_2 adsorption isotherms for NH_3-loaded samples: (a) **Eu-II-NH$_3$-14%** and (b) **Yb-III-NH$_3$-14%**.

Figure S22. H_2O vapor isotherms for: (a) La[O_3P-C_6H_3(COO)(COOH)(H_2O)$_2$] (**La-I**), (b) Eu_2{[O_3P-C_6H_3(COO)(COOH)]$_2$(H_2O)$_4$}·2H_2O **(Eu-II)** and (c) Yb[O_3P-C_6H_3(COO)(COOH)(H_2O)] **(Yb-III)**.

Figure S23. H_2O vapor isotherms for NH_3-loaded derivatives: (a) **Eu-II-NH$_3$-14%** and (b) **Yb-III-NH$_3$-14%**.

Figure S24. SEM images of (a) **Eu-II,** (b) **Eu-II-NH_3-28%_R,** (c) **Yb-III** and (d) **Yb-III-NH_3-28%_R**.

Table S1. Structural characteristics and proton conductivity values of reported metal di-, tricarboxylate and triphosphonates.

Table S2. H-bond interactions for La[O_3P-C_6H_3(COO)(COOH)(H_2O)$_2$], **La-I**.

Table S3. H-bond interactions for Eu_2{[O_3P-C_6H_3(COO)(COOH)]$_2$(H_2O)$_4$}·$2H_2O$, **Eu-II**.

Table S4. H-bond interactions for Yb[O_3P-C_6H_3(COO)(COOH)(H_2O)], **Yb-III**.

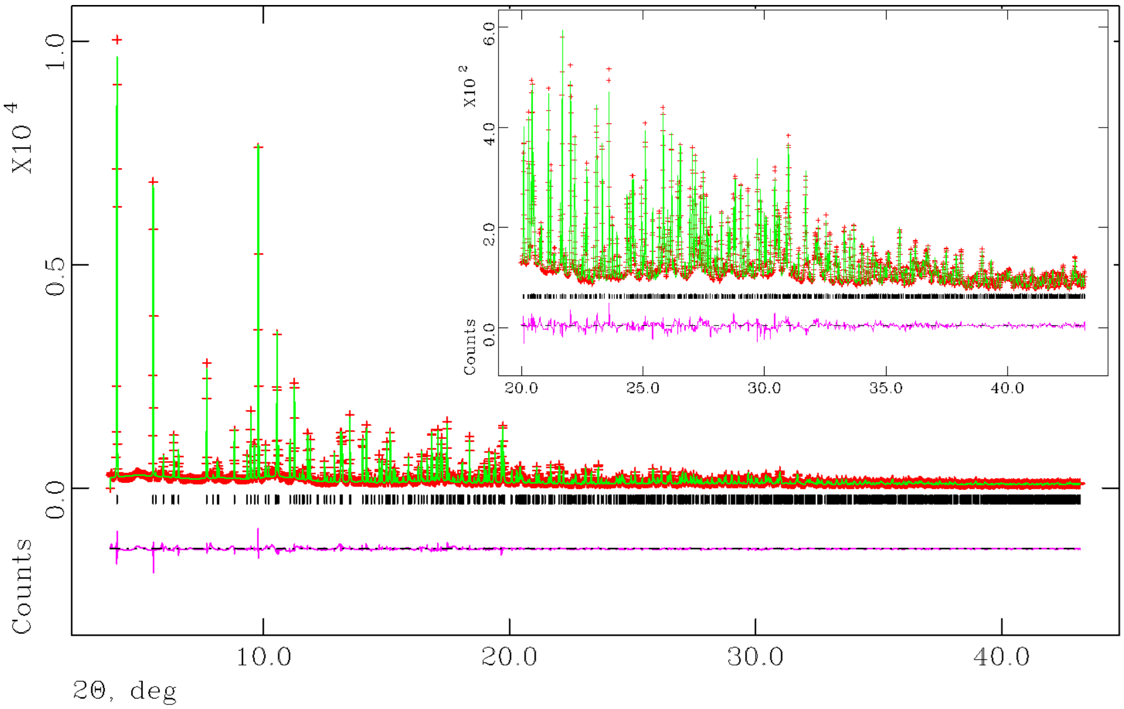

Figure S1. Rietveld plot for La[O_3P-C_6H_3(COO)(COOH)(H_2O)$_2$], **La-I**.

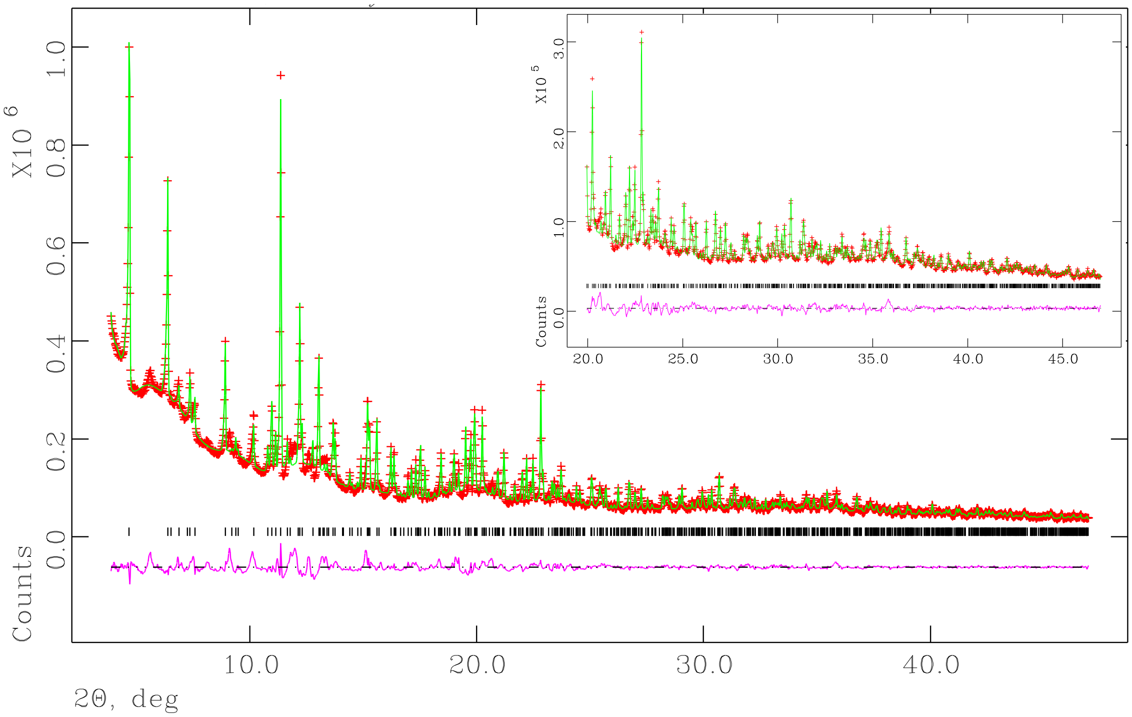

Figure S2. Rietveld plot for Pr[O_3P-C_6H_3(COO)(COOH)(H_2O)$_2$], **Pr-I**.

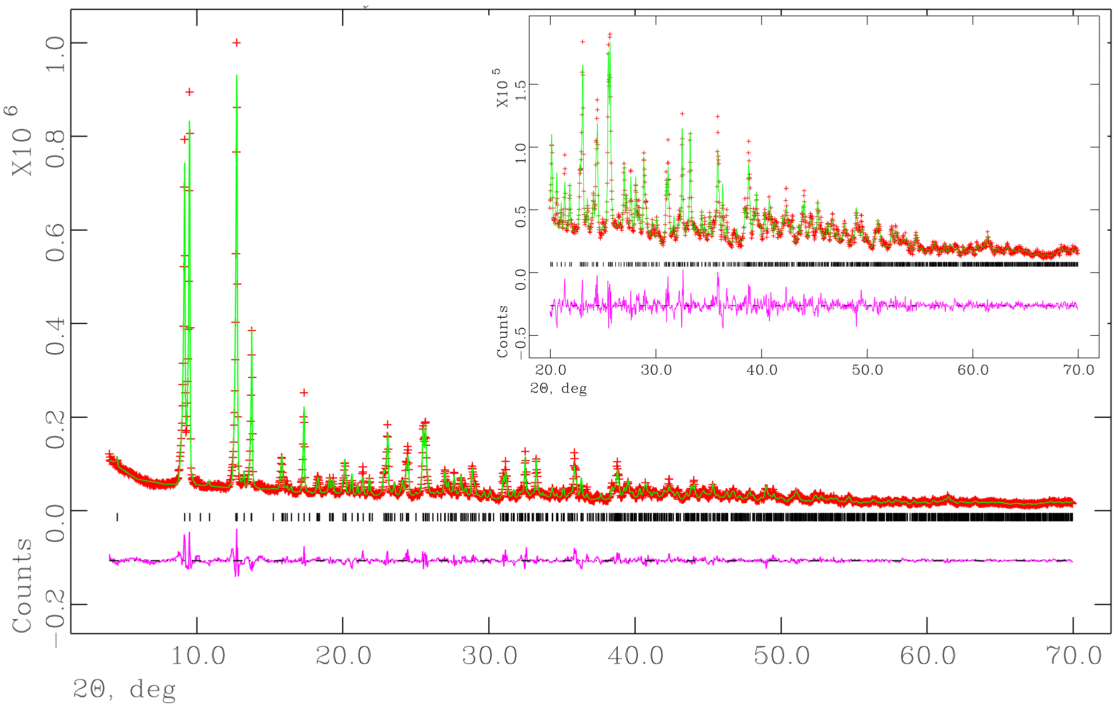

Figure S3. Rietveld plot for $La_2\{[O_3P\text{-}C_6H_3(COO)(COOH)]_2(H_2O)_4\}\cdot 2H_2O$, **La-II**.

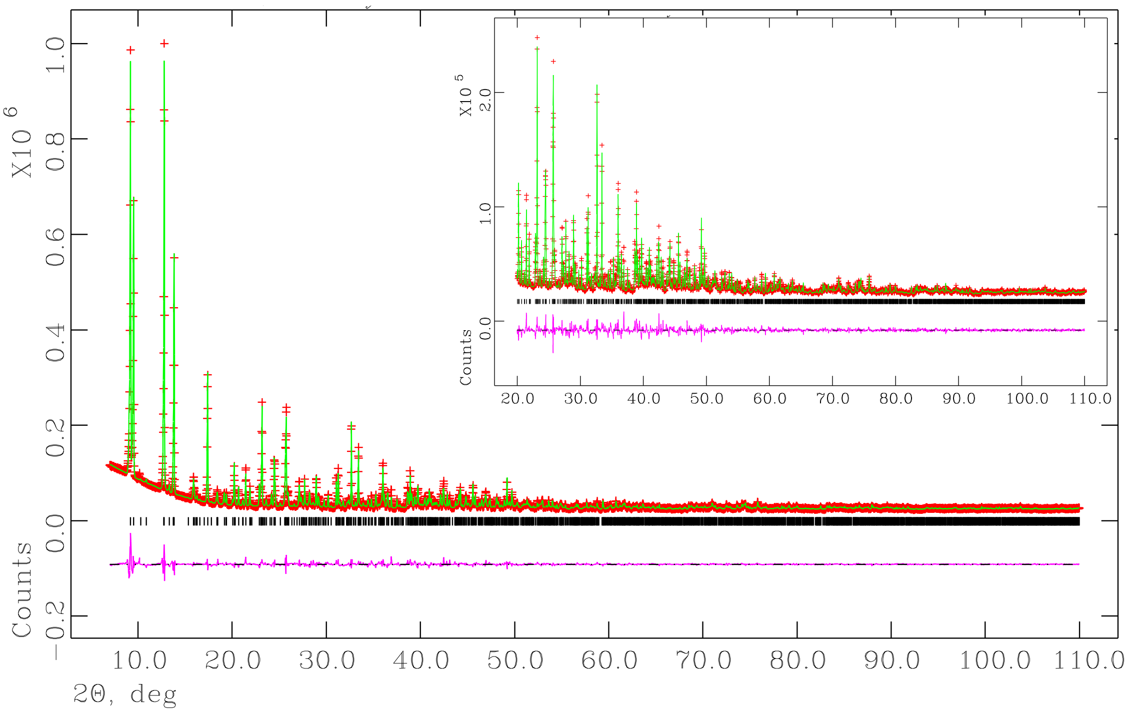

Figure S4. Rietveld plot for $Pr_2\{[O_3P\text{-}C_6H_3(COO)(COOH)]_2(H_2O)_4\}\cdot 2H_2O$, **Pr-II**.

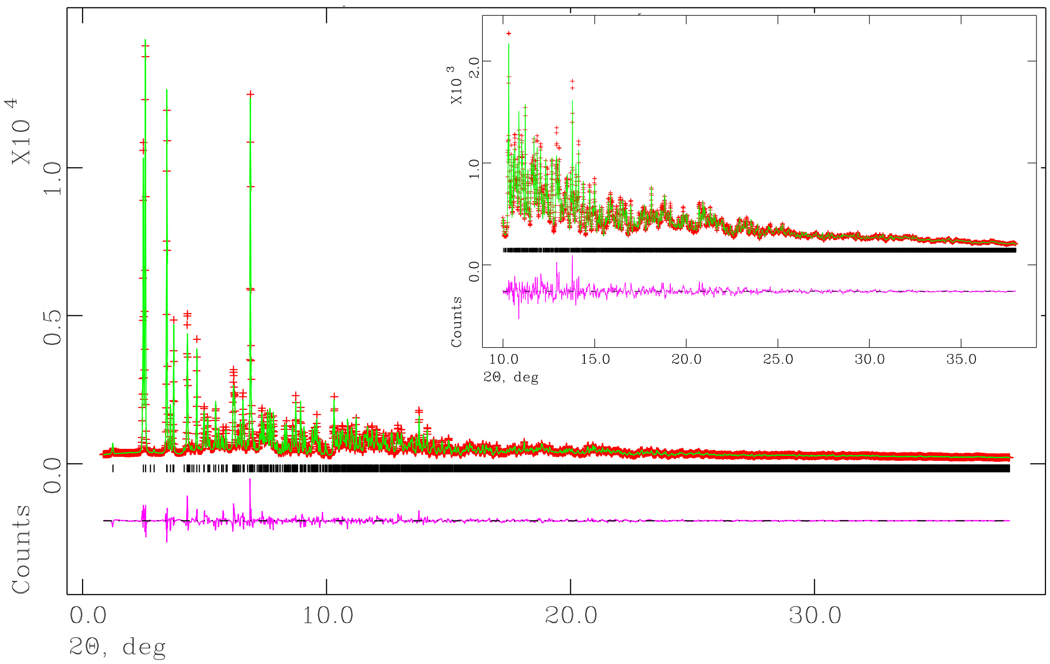

Figure S5. Rietveld plot for $Eu_2\{[O_3P\text{-}C_6H_3(COO)(COOH)]_2(H_2O)_4\}\cdot 2H_2O$, **Eu-II**.

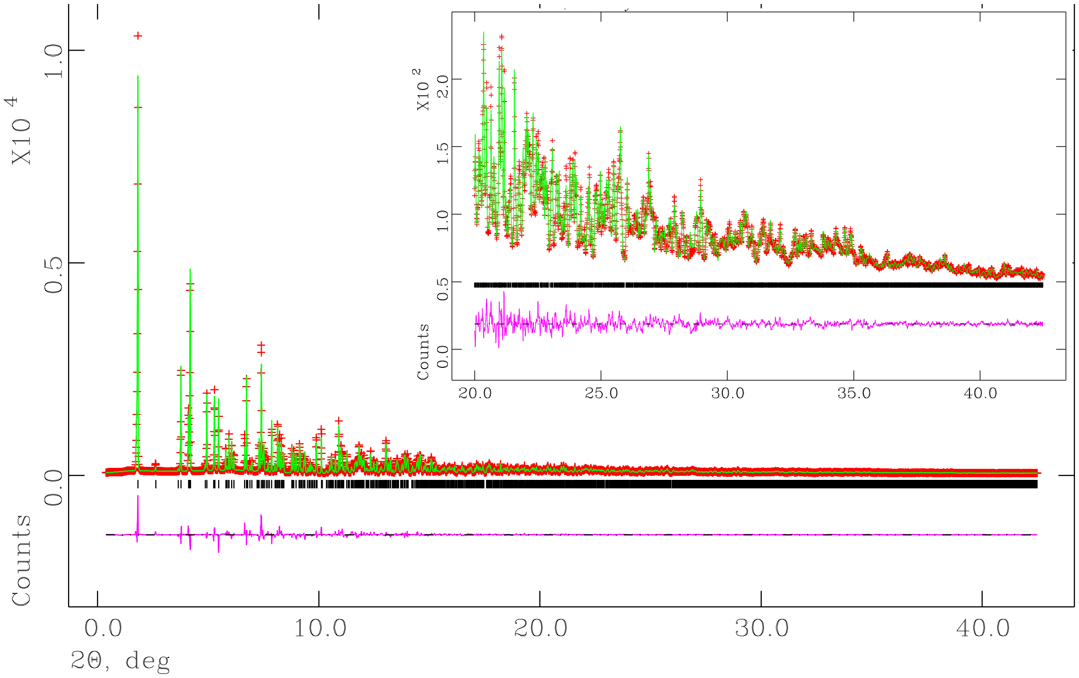

Figure S6. Rietveld plot for $Yb[O_3P\text{-}C_6H_3(COO)(COOH)(H_2O)]$, **Yb-III**.

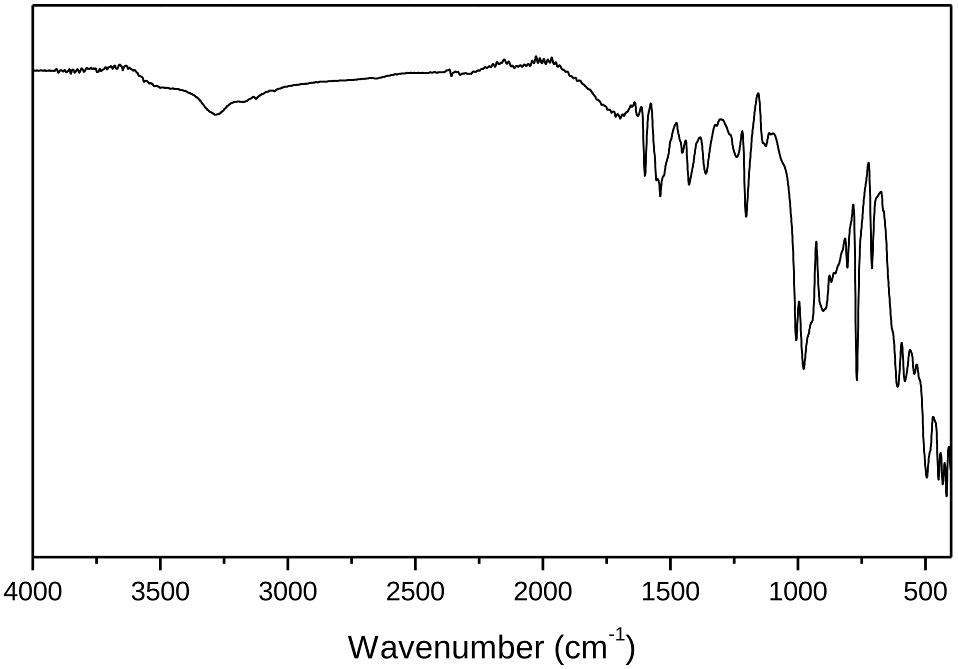

Figure S7. FT-IR spectrum for as-synthesized **La-I**.

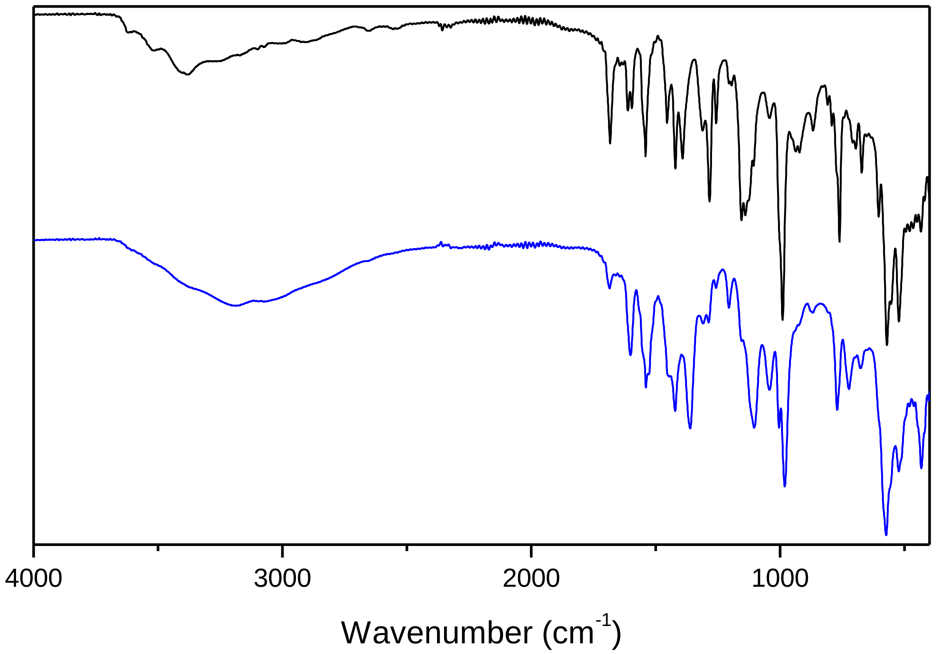

Figure S8. FT-IR spectra for as-synthesized **Eu-II** (black) and upon ammonia vapors exposure, **Eu-II-NH_3-14%** (blue).

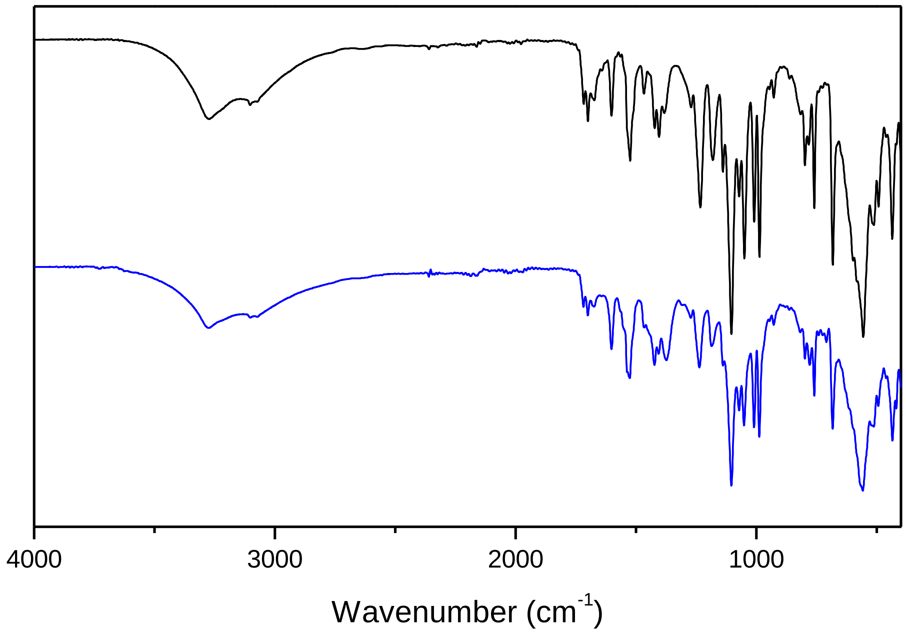

Figure S9. FT-IR spectra for as-synthesized **Yb-III** (black) and **Yb-III-NH$_3$-14%** (blue).

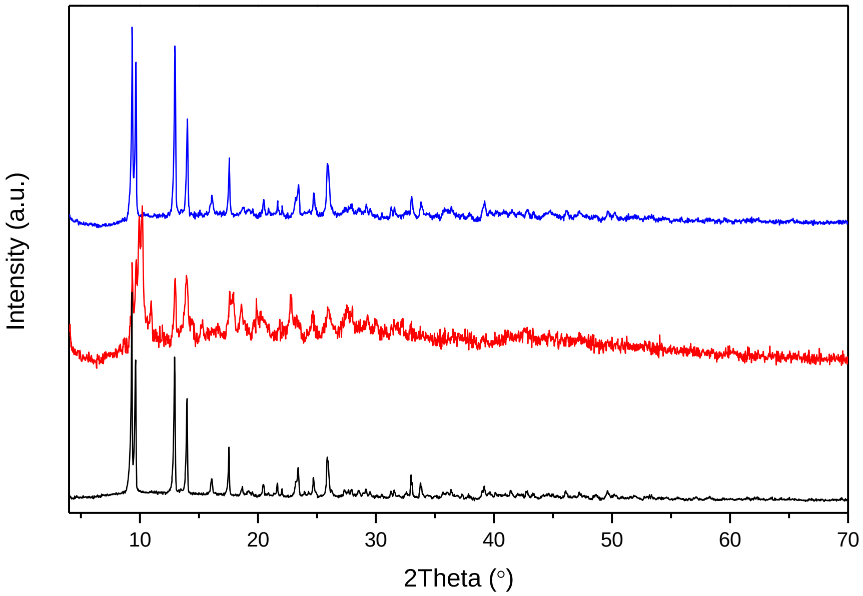

Figure S10. X-ray powder diffraction patterns for **Eu-II** compound as-synthesized (black), heated at 200 ºC (red) and rehydrated (blue) at RT.

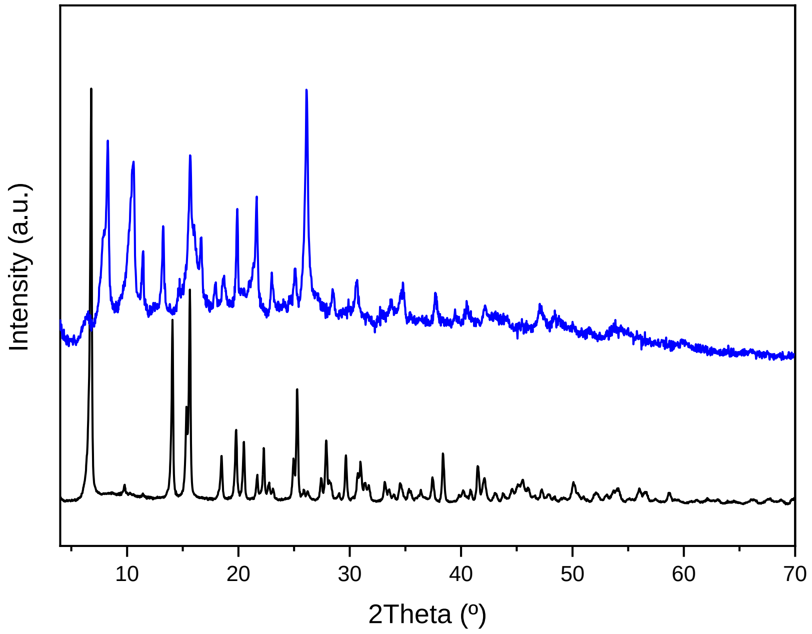

Figure S11. X-ray powder diffraction patterns for **Yb-III** as-synthesized (black) and after heated at 300 ºC and rehydrated in K_2SO_4 saturated solution atmosphere at RT (blue).

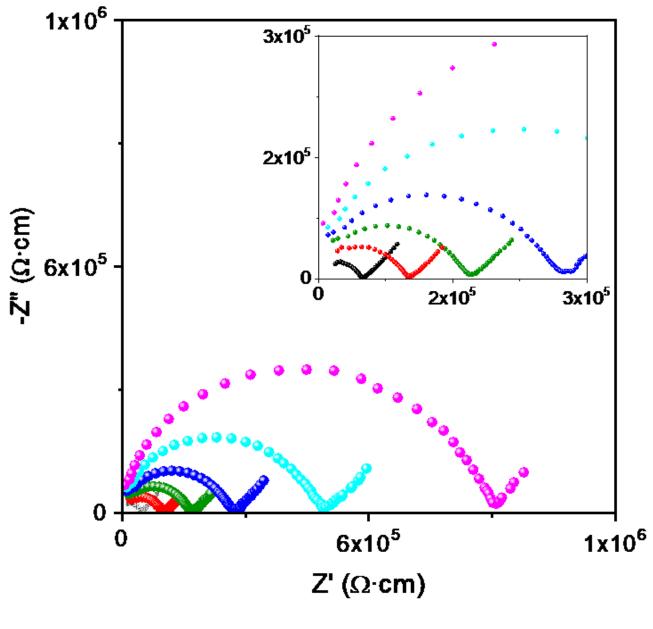

Figure S12. Complex impedance plane plots for **La-I** at 95% RH and different temperatures: 80 (black), 70 (red), 60 (green), 50 (blue), 40 (cyan) and 30 ºC (magenta).

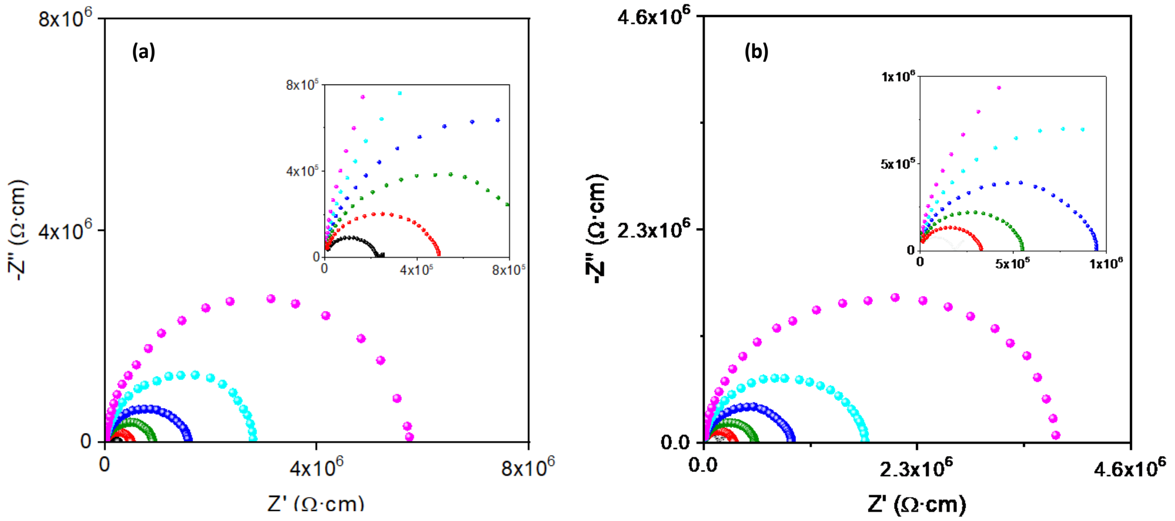

Figure S13. Complex impedance plane plots for (a) **La-II** and (b) **Eu-II** at 95% RH and different temperatures: 80 (black), 70 (red), 60 (green), 50 (blue), 40 (cyan) and 30 ºC (magenta).

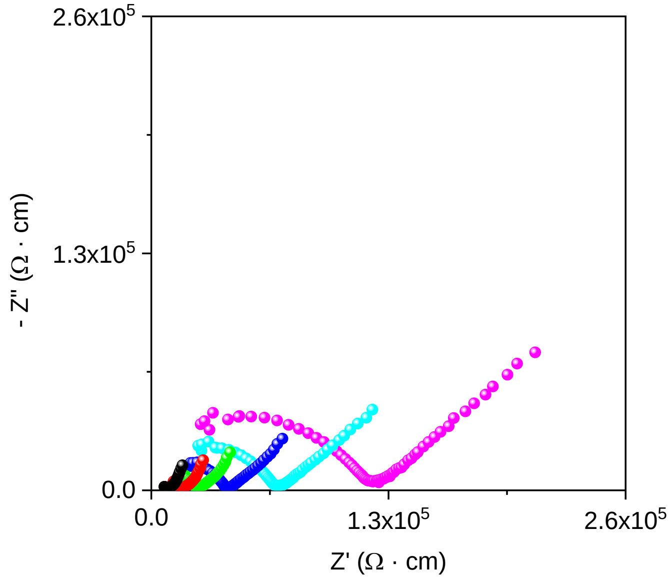

Figure S14. Complex impedance plane plots for **Yb-III** at 95% RH and different temperatures: 80 (black), 70 (red), 60 (green), 50 (blue), 40 (cyan) and 30 ºC (magenta).

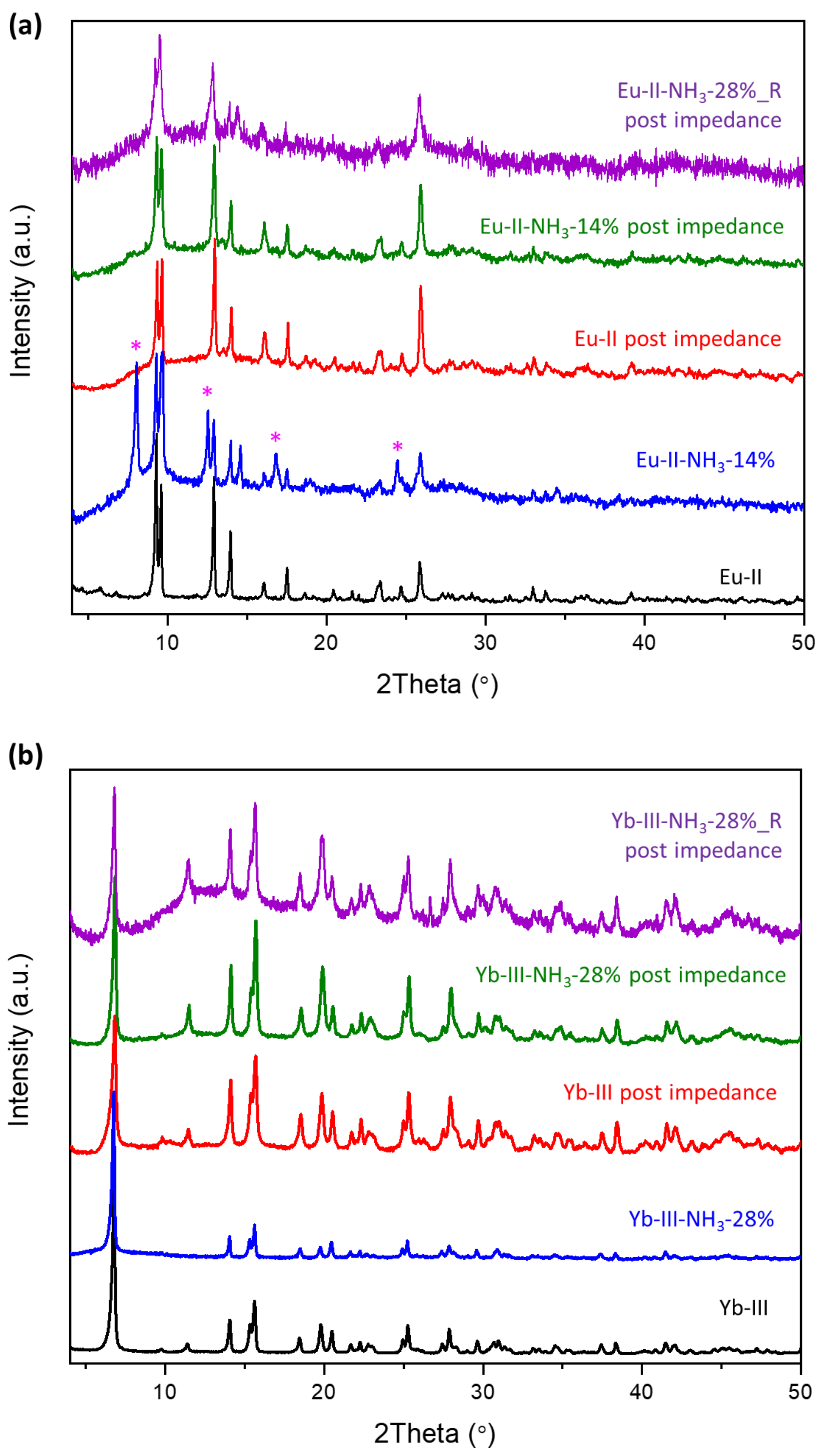

Figure S15. X-ray powder diffraction patterns for (a) **Eu-II** and (b) **Yb-III** compounds. *Diffraction peaks corresponding to a new NH_3-containing **Eu-II** phase.

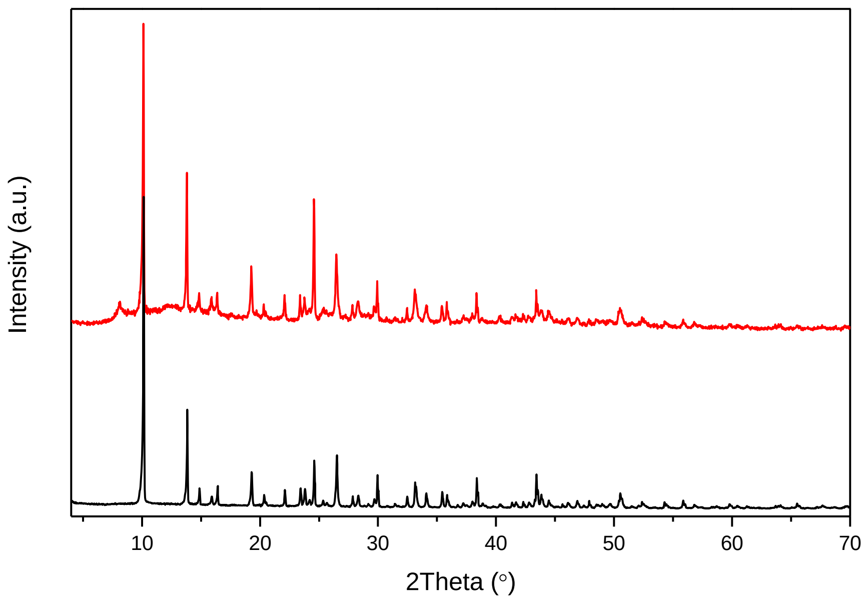

Figure S16. XRPD patterns for as-synthetized (black) and post impedance measurement samples (red) for **La-I.**

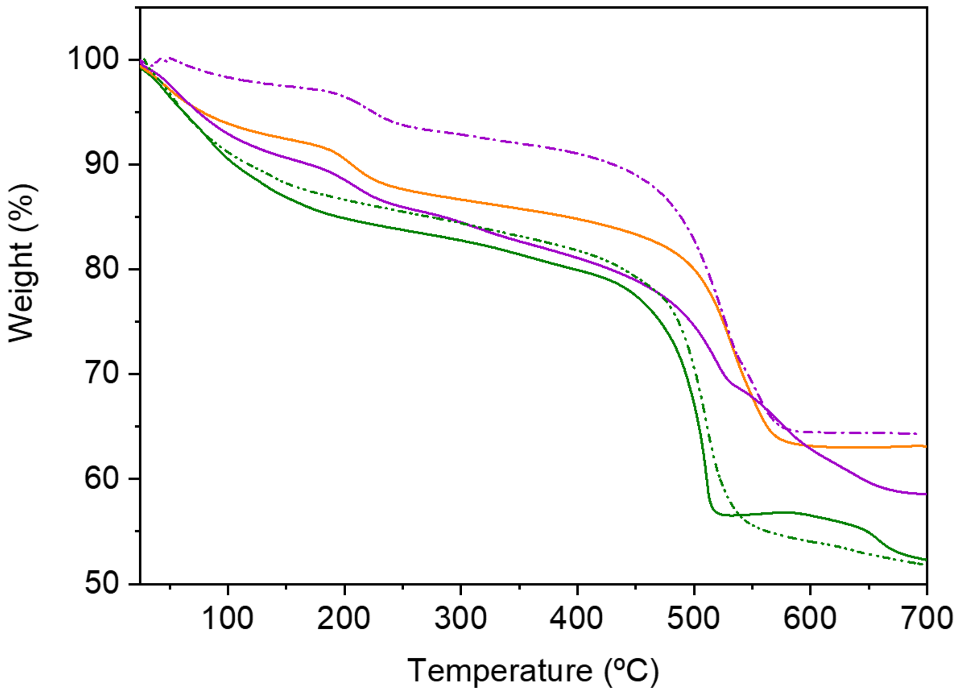

Figure S17. TG curves for **Eu-II-NH$_3$-14%** (solid green), **Eu-II-NH$_3$-28%_R** (dash dot green), **Yb-III-NH$_3$-14%** (solid orange), **Yb-III-NH$_3$-28%** (solid purple) and **Yb-III-NH$_3$-28%_R** (dash dot purple) derivatives.

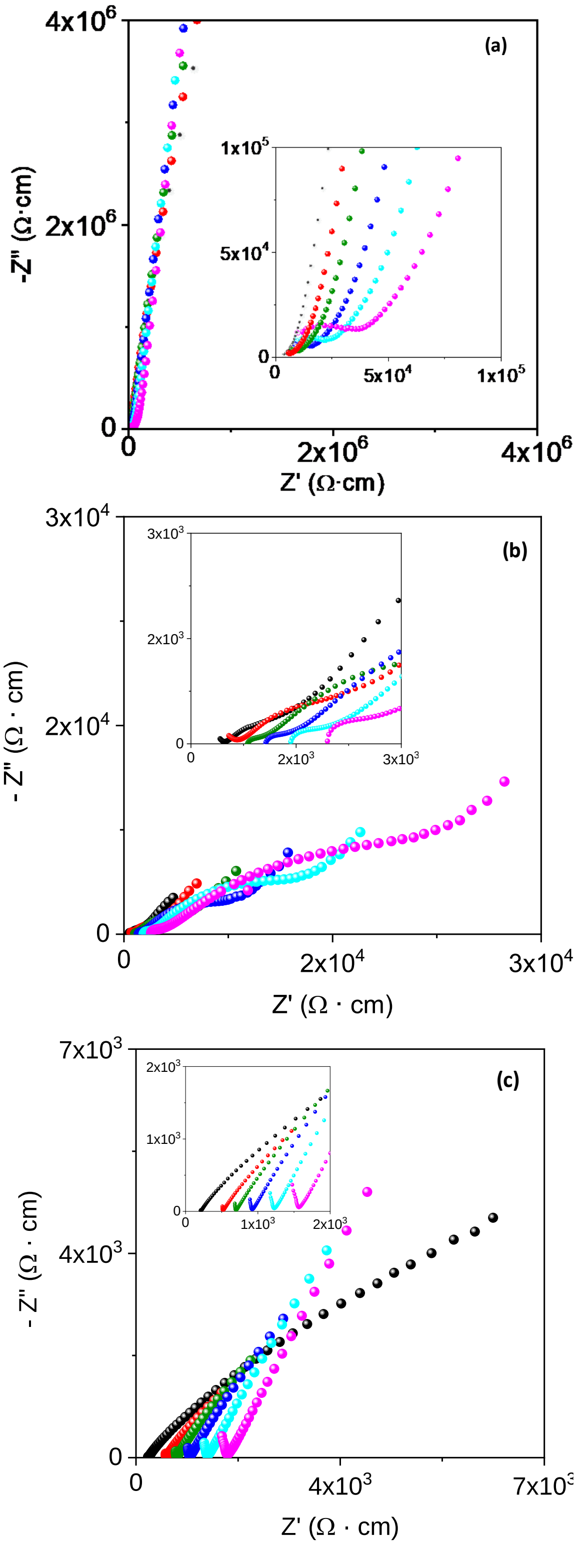

Figure S18. Complex impedance plane plots at 95% RH for (a) **Eu-II-NH$_3$-14%**, (b) **Yb-III-NH$_3$-14%** and (c) **Yb-III-NH$_3$-28%** at different temperatures: 80 (black), 70 (red), 60 (green), 50 (blue), 40 (cyan) and 30 ºC (magenta).

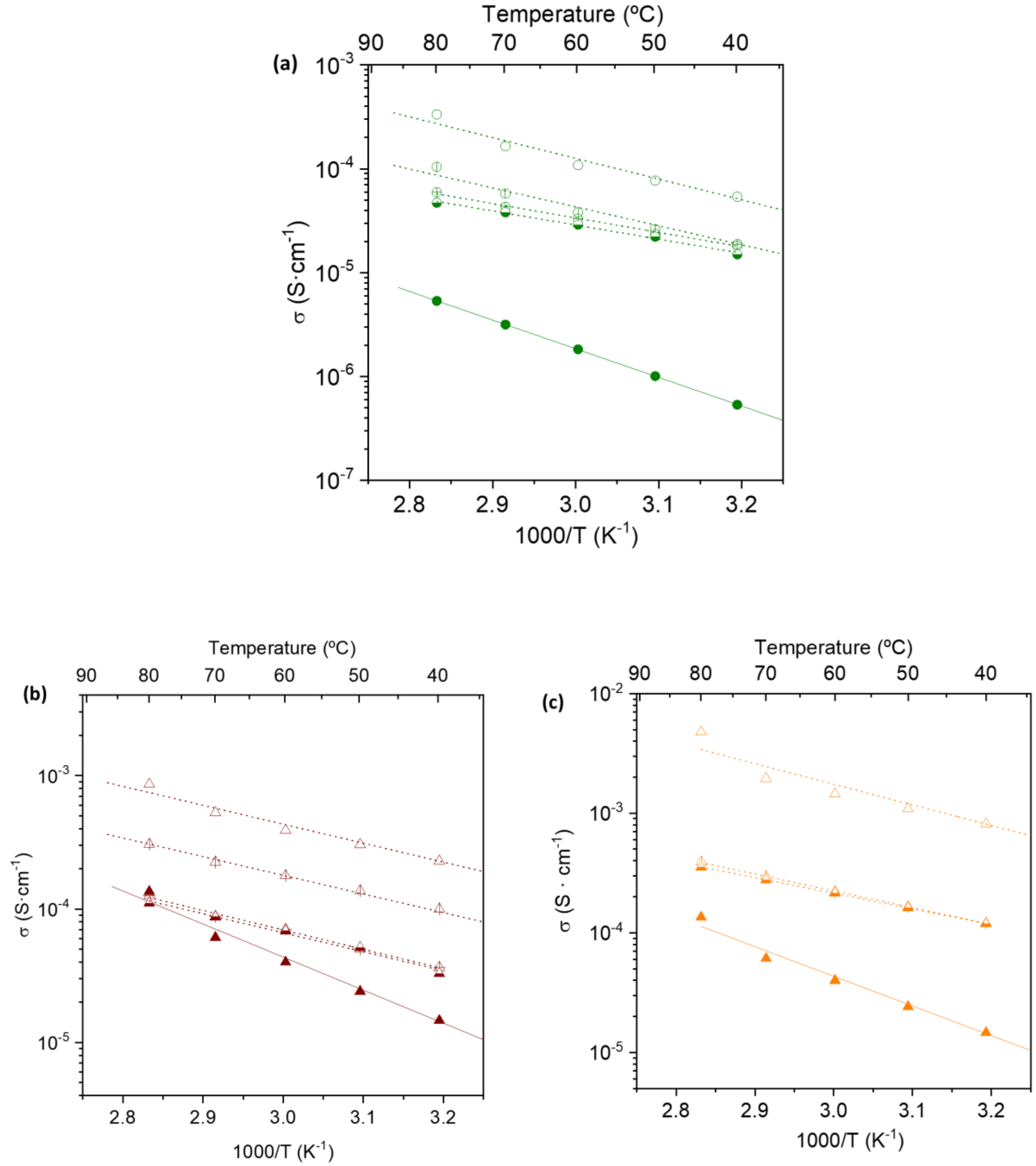

Figure S19. Arrhenius plots for NH_3-containing derivatives at 95% RH and different EIS measurements: (a) **Eu-II** as-synthetized (●), **Eu-II-NH_3-14%** 1st (○), 2nd (⦶), 3rd (⊕) and 4rd cycles (◒); (b) **Yb-III** as-synthetized (▲), **Yb-III-NH_3-14%** 1st (△), 2nd (△), 3rd (△) and 4rd cycles (△); (c) **Yb-III** as-synthetized (▲), **Yb-III-NH_3-28%** 1st (△), 2nd (△) and 3rd cycles (△).

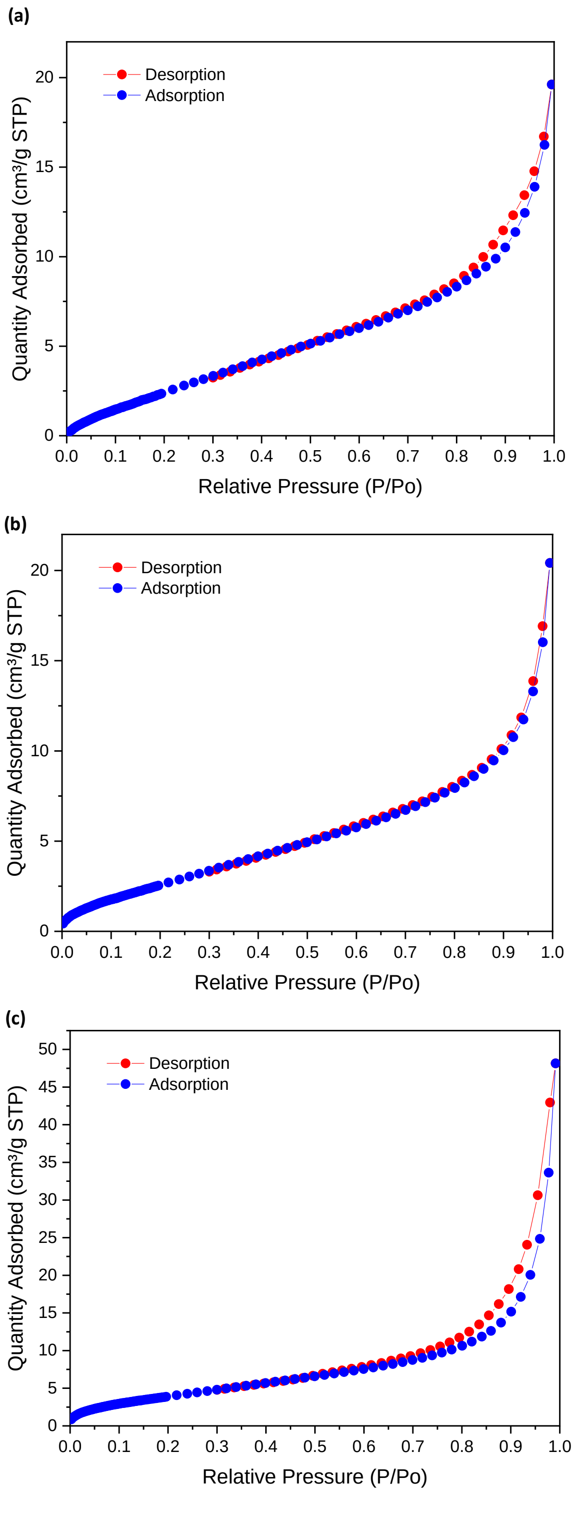

Figure S20. N_2 adsorption isotherms for: (a) La[O_3P-C_6H_3(COO)(COOH)(H_2O)$_2$] (**La-I**), (b) Eu_2{[O_3P-C_6H_3(COO)(COOH)]$_2$(H_2O)$_4$}·2H_2O (**Eu-II**) and (c) Yb[O_3P-C_6H_3(COO)(COOH)(H_2O)] (**Yb-III**).

(a)

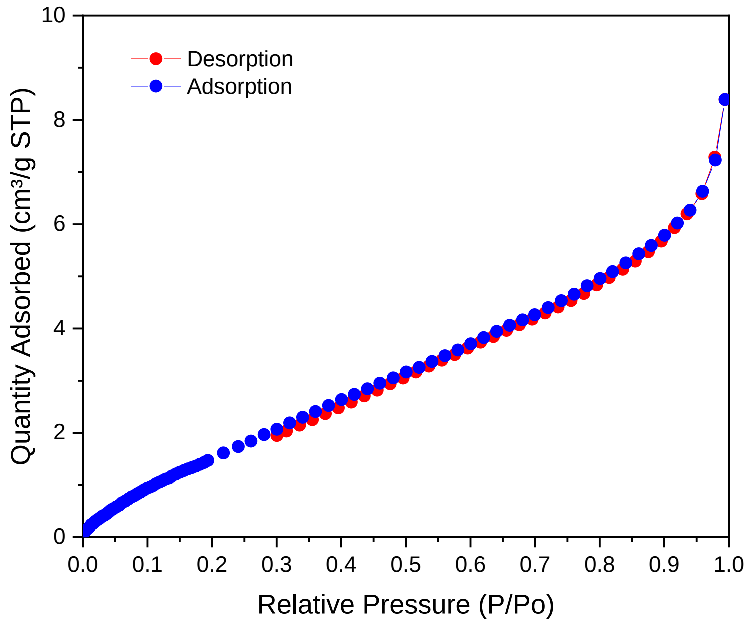

(b)

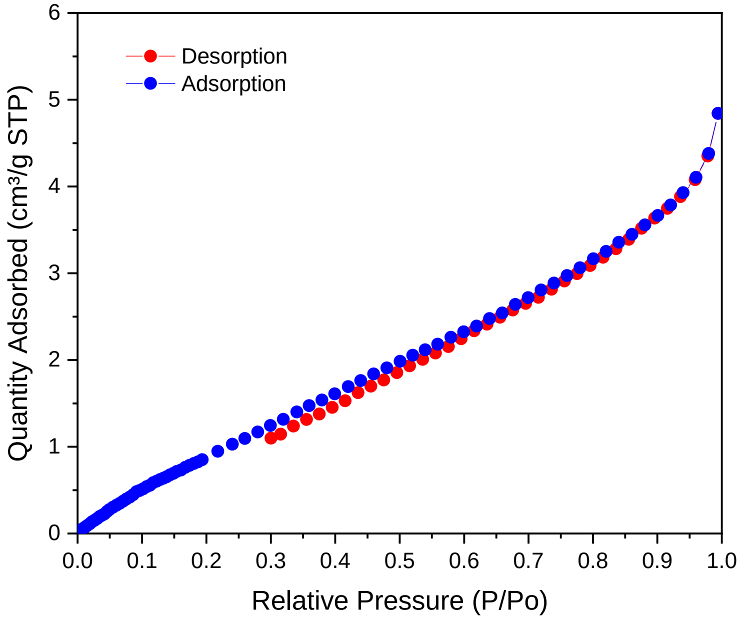

Figure S21. N_2 adsorption isotherms for NH_3-loaded samples: (a) **Eu-II-NH_3-14%** and (b) **Yb-III-NH_3-14%**.

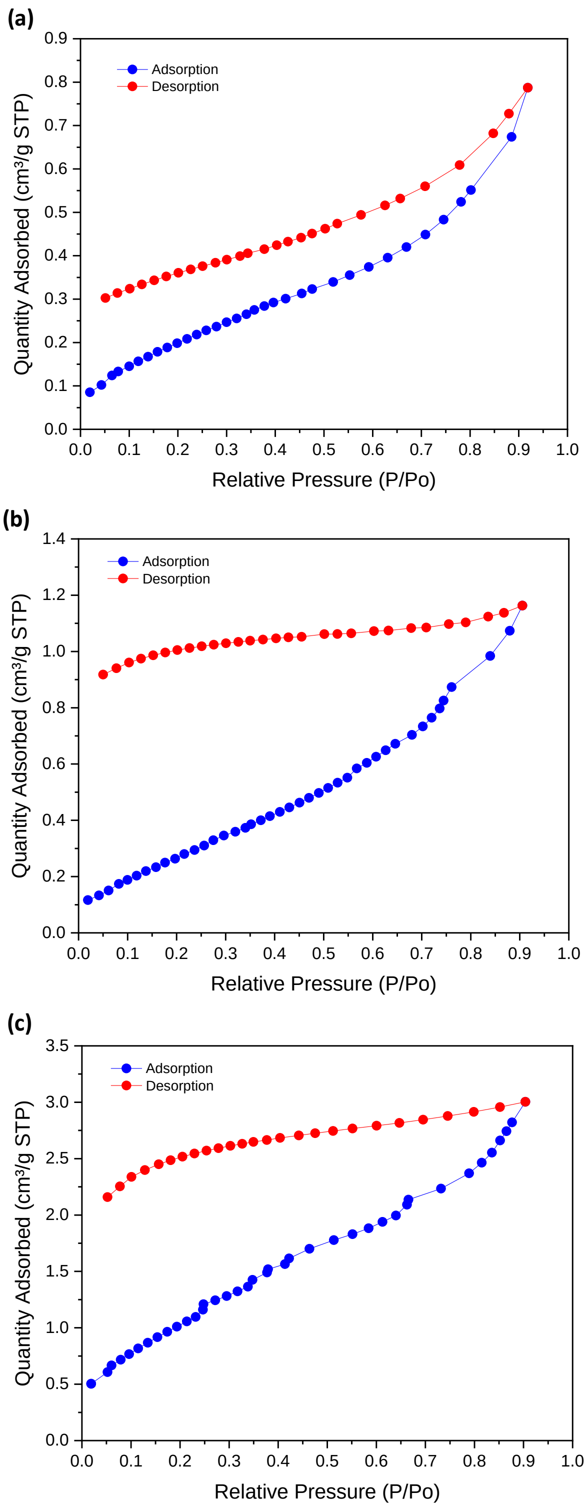

Figure S22. H_2O vapor isotherms for: (a) La[O_3P-C_6H_3(COO)(COOH)(H_2O)$_2$] (**La-I**), (b) Eu_2{[O_3P-C_6H_3(COO)(COOH)]$_2$(H_2O)$_4$}·$2H_2O$ **(Eu-II)** and (c) Yb[O_3P-C_6H_3(COO)(COOH)(H_2O)] **(Yb-III)**.

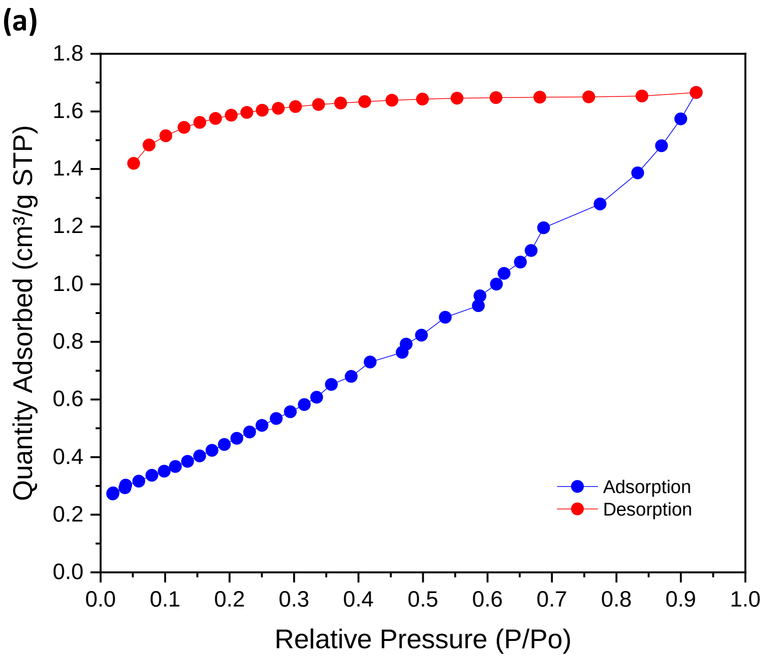

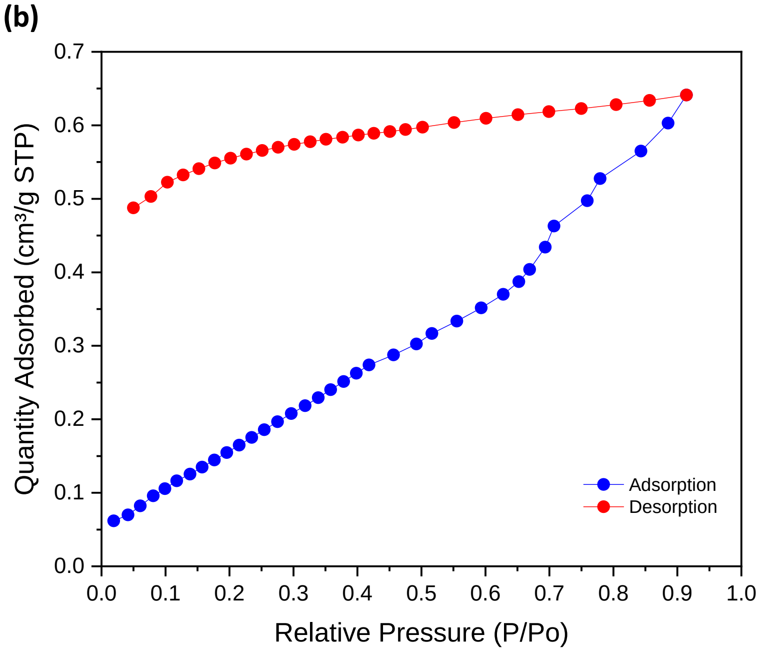

Figure S23. H_2O vapor isotherms for NH_3-loaded derivatives: (a) **Eu-II-NH_3-14%** and (b) **Yb-III-NH_3-14%**.

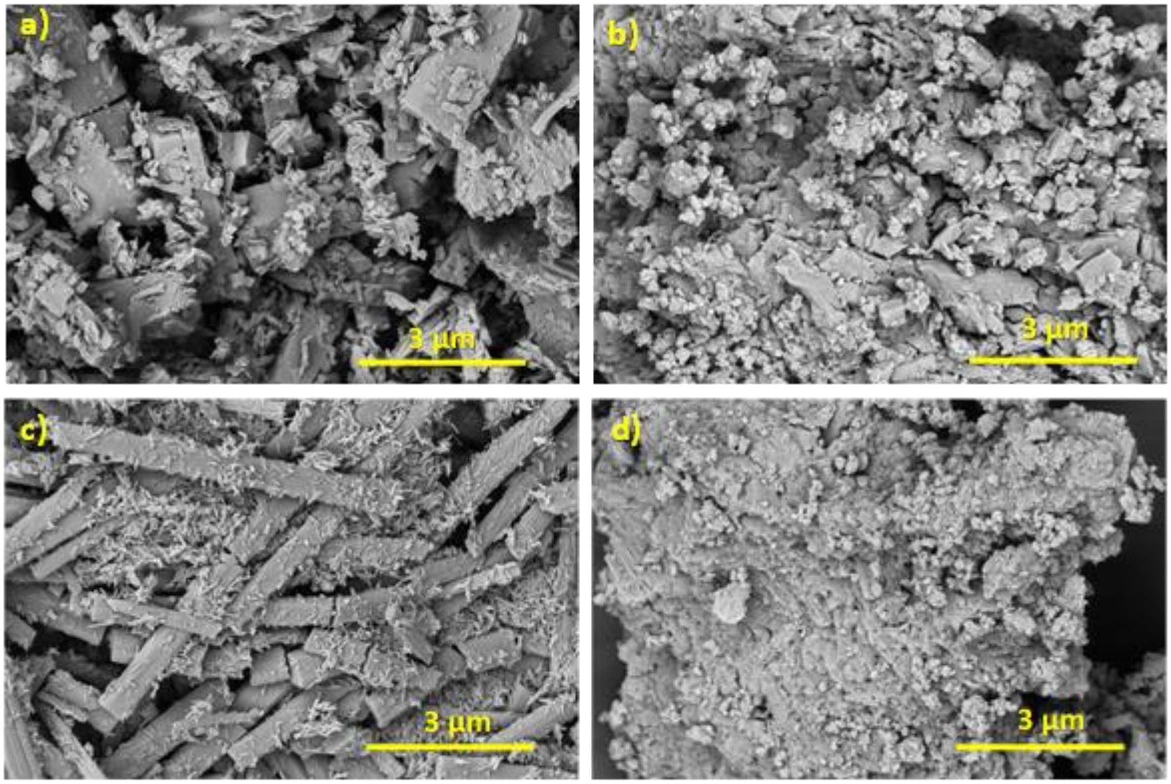

Figure S24. SEM images of (a) **Eu-II,** (b) **Eu-II-NH_3-28%_R,** (c) **Yb-III** and (d) **Yb-III-NH_3-28%_R**.

Table S1. Structure and proton conductivity of selected carboxylate and/or phosphonate-containing coordination polymers as related to the title compounds.

Organic ligand	Compound	Dimensionality	σ ($S\cdot cm^{-1}$)/E_a (eV)	Ref.
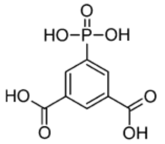	$Ca_2[(HO_3PC_6H_3COOH)_2]_2[(HO_3PC_6H_3(COO)_2H)(H_2O)_2]\cdot 5H_2O$	Pillared layered	**$5.7\cdot 10^{-4}$/0.32**	1
	$Ca_2[(HO_3PC_6H_3COOH)_2]_2[(HO_3PC_6H_3(COO)_2H)]\cdot 16H_2O\cdot 7NH_3$	1D	**$6.6\cdot 10^{-3}$/0.40** (24 ºC, 98% RH)	
	$Ca_2(H_2O)[H(OOC)_2C_6H_3\text{-}PO_3H]_2$	Pillared layered	--	2
	$Sm_2\{[(O_3P\text{-}C_6H_3(COO)(COOH)]_2(H_2O)_4\}\cdot 2H_2O$	3D (1D channels)	--	
	$Ba_2(H_2O)_3(OOC)_2C_6H_3\text{-}PO_3$	Pillared layered	--	
	$[Cu_3(H_2O)(H(OOC)_2C_6H_3\text{-}PO_3)_2]$ $2H_2O$	Layered	--	
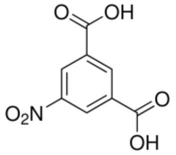	$\{H[(N(CH_3)_4)_2][Gd_3(NPA)_6]\}\cdot 3H_2O$	3D	**$7.17\cdot 10^{-2}$/0.13** (75 ºC, 98% RH)	3
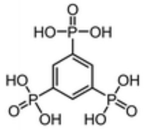	$Ba(C_6H_3(PO_3H)_2(PO_3H_2)$ (H_2O)	Pillared layered	-	4
	$Cu_6[C_6H_3(PO_3)_3]_2(H_2O)_8\}\cdot 5.5H_2O$	3D	-	5
	$Zn_3(C_6H_3(PO_3)_3)(H_2O)_2\cdot 2$ H_2O	2D	**$3.5\cdot 10^{-5}$/0.17** (25 °C, 98% RH)	6
	$[La(C_6H_3(PO_3H)_3)(H_2O)_2]$	2D	-	7
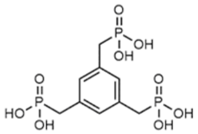	$[Cu_3(C_6H_3(CH_2PO_3)_3)(H_2O)_{3.6}]\cdot H_2O$	3D	-	8
	$Ln_2(C_6H_3(CH_2PO_3H)_3)_2]\cdot H_2O$ [Ln^{3+}=Eu^{3+}, Gd^{3+}, Tb^{3+}, Dy^{3+}, Ho^{3+}, Er^{3+}, Tm^{3+}, Yb^{3+}	2D	-	9

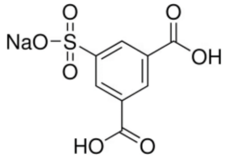	$[Ho(C_6H_3(SO_3H)(COO)_2)(H_2O)_5]\cdot 3H_2O$	1D	**8.2·10^{-4}/0.46**<br>(70 °C, 98% RH)	10
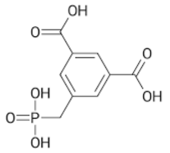	$[(Me_2NH_2)[Eu(C_6H_3(CH_2PO_3H)(COO)_2)]$	2D	**1.25·10^{-3}/0.38**<br>(150 °C, SC)<br>**3.76·10^{-3}/0.38**<br>(100 °C, 98%RH)	11
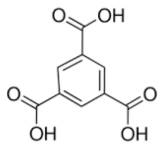	Cu-BTC	3D	**1·10^{-7}**<br>(25 °C, 100%RH)	12
	Cu-BTC-Lys (10 wt%)	3D	**1.67·10^{-6}/0.84**<br>(25 °C, 100%RH)	
	Cu-BTC-Lys (43 wt%)	3D	**1.2·10^{-3}/0.25**<br>(25 °C, 100%RH)	

References:

1. M. Bazaga-García, R.M.P. Colodrero, M. Papadaki, P. Garczarek, J. Zoń, P. Olivera-Pastor, E.R. Losilla, L. León-Reina, M.A.G. Aranda, D. Choquesillo-Lazarte, K.D. Demadis, A. Cabeza. Guest Molecule-Responsive Functional Calcium Phosphonate Frameworks for Tuned Proton Conductivity. J. Am. Chem. Soc. 2014, 136, 5731−5739.

2. S. Bauer, N. Stock. Synthesis and characterization of four new metal 5-phosphonoisophthalates discovered by high-throughput experimentation. Journal of Solid State Chemistry 2007, 180, 3111–3120.

3. X.S. Xing, Z.H. Fu, N.N. Zhang, X.Q. Yu, M.S. Wang, G.C. Guo. High proton conduction in an excellent water-stable gadolinium metal–organic framework. Chem Commun 2019, 55,1241–1244.

4. B.S. Gelfand, J.M. Taylor, G.K.H. Shimizu. Extracting structural trends from systematic variation of phosphonate/phosphonate monoester coordination polymers. CrystEngComm, 2017,19, 3727-3736.

5. D. Kong, J. Zoñ, J.r McBee, A. Clearfield. Rational Design and Synthesis of Porous Organic−Inorganic Hybrid Frameworks Constructed by 1,3,5-Benzenetriphosphonic Acid and Pyridine Synthons. Inorg. Chem. 2006, 45, 3, 977–986.

6. J. M. Taylor, R. K. Mah, I. L. Moudrakovski, C. I. Ratcliffe, R. Vaidhyanathan, G. K. H. Shimizu. Facile Proton Conduction via Ordered Water Molecules in a Phosphonate Metal−Organic Framework. J. Am. Chem. Soc. 2010, 132, 14055–14057.

7. T. Araki, A. Kondo, K. Maeda, Chem. Commun. 2013, 49, 552–554.

8. C.-I. Yang, Y.-T. Song, Y.-J. Yeh, Y.-H. Liu, T.-W. Tseng, K.-L. Lu, CrystEng-Comm 2011, 13, 2678–2686

9. F.A. Almeida Paz, S.M.F. Vilela, J.P.C. Tomé. Layered Metal–Organic Frameworks Based on Octahedral Lanthanides and a Phosphonate Linker: Control of Crystal Size. Cryst. Growth Des. 2014, 14, 10, 4873–4877

10. S.S. Wang, X.Y. Wu, Z. Li, C.Z. Lu. Designed synthesis of a proton-conductive Ho-MOF with reversible dehydration and hydration. Dalton Trans 2019, 48, 9930–9934

11. Y.S. Wei, X.P. Hu, Z. Han, X.Y. Dong, S.Q. Zang, T.C. Mak. Unique proton dynamics in an efficient MOF-based proton conductor. J Am Chem Soc 2017, 139, 3505–3512

12. Y. Gao, B. Liu, H. Xu, C. Shi, N. Yan, S. Wang, R. Jiang. Enhanced proton conductivity in a Cu-BTC thin-film membrane through lysine incorporation and a mixed matrix membrane. New J. Chem., 2023, 47, 13638

Table S2. H-bond interactions for La[O_3P-C_6H_3(COO)(COOH)(H_2O)$_2$], **La-I**.

**D-H···A**	**D···A (Å)**
O7 ···O2	2.463(15)
Ow1 ···O2	2.972(35)
Ow2 ···O2	2.88(4)
Ow1 ···O4	2.774(23)
Ow2 ···O4	2.577(24)

Table S3. H-bond interactions for Eu_2{[O_3P-C_6H_3(COO)(COOH)]$_2$(H_2O)$_4$}·$2H_2O$, **Eu-II**.

**D-H···A**	**D···A (Å)**	**D-H···A**	**D···A (Å)**
O11····O14	2.620(33)	Ow4 ···O11	2.789(25)
Ow1 ···O8	2.68(4)	Ow4 ···Ow5	2.42(4)
Ow1 ···Ow4	2.551(25)	Ow4····Ow6	2.93(4)
Ow2 ···Ow4	2.727(26)	Ow5····O11	2.91(4)
Ow3···O5	2.67(4)	Ow6···O6	2.88(4)
Ow4 ···O8	2.57(4)	Ow6···O8	2.67(4)

Table S4. H-bond interactions for Yb[O_3P-C_6H_3(COO)(COOH)(H_2O)], **Yb-III**.

**D-H···A**	**D···A (Å)**
O4 ···O6	2.908(17)
Ow1 ···O1	2.444(21)
Ow1 ···O3	2.974(21)
Ow1 ···O7	2.824(20)
